# Antibacterial Components and Modes of the Methanol-Phase Extract from *Commelina communis* Linn

**DOI:** 10.3390/plants12040890

**Published:** 2023-02-16

**Authors:** Yue Liu, Yingping Tang, Shunlin Ren, Lanming Chen

**Affiliations:** 1 Key Laboratory of Quality and Safety Risk Assessment for Aquatic Products on Storage and Preservation, Ministry of Agriculture and Rural Affairs of the People’s Republic of China, Shanghai 201306, China; 2 College of Food Science and Technology, Shanghai Ocean University, Shanghai 201306, China; 3 Department of Internal Medicine, Virginia Commonwealth University/McGuire VA Medical Centre, Richmond, VA 23298, USA

**Keywords:** antibacterial mechanism, antibacterial substances, *Commelina communis* Linn, traditional Chinese herb, pathogenic bacteria

## Abstract

Infectious diseases caused by pathogenic bacteria severely threaten human health. Traditional Chinese herbs are potential sources of new or alternative medicine. In this study, we analyzed for the first time antibacterial substances in the methanol-phase extract from a traditional Chinese herb—*Commelina communis* Linn—which showed an inhibition rate of 58.33% against 24 species of common pathogenic bacteria. The extract was further purified using preparative high-performance liquid chromatography (Prep-HPLC), which generated four single fragments (Fragments 1 to 4). The results revealed that Fragment 1 significantly increased bacterial cell surface hydrophobicity and membrane permeability and decreased membrane fluidity, showing disruptive effects on cell integrity of Gram-positive and Gram-negative bacteria, such as *Bacillus cereus*, *Enterococcus faecalis*, *Staphylococcus aureus*, and *Salmonella enterica* subsp., compared to the control groups (*p* < 0.05). In sum, 65 compounds with known functions in Fragment 1 were identified using liquid chromatography and mass spectrometry (LC-MS), of which quercetin-3-o-glucuronide was predominant (19.35%). Comparative transcriptomic analysis revealed multiple altered metabolic pathways mediated by Fragment 1, such as inhibited ABC transporters, ribosome, citrate cycle and oxidative phosphorylation, and upregulated nitrogen metabolism and purine metabolism, thereby resulting in the repressed bacterial growth and even death (*p* < 0.05). Overall, the results of this study demonstrate that Fragment 1 from *C. communis* Linn is a promising candidate against common pathogenic bacteria.

## 1. Introduction

*Commelina communis* Linn (known as herba commelinae) is an annual herb that grows in tropical and subtropical regions of the world [1]. This plant belongs to the phylum of Angiospermae, class Monocotyledoneae, suborder Commelinineae, family Commelinaceae, and genus Commelina. *C. communis* Linn is often used as a febrifuge or diuretic in Chinese folk medicine to treat high fever, sore throat, edema oliguria, astringent pain, and bloated furuncle poison [2]. The herb was recorded in the *Pharmacopoeia of the People’s Republic of China* in 1977. Recent research has also shown that *C. communis* Linn contains alpha-glucosidase-inhibiting polyhydroxyalkaloids and could be used to therapy non-insulin-dependent diabetes [3]. Nevertheless, current literature on bacteriostatic activity of *C. communis* Linn is rare. To the best of our knowledge, only Tang and colleagues [4] reported that the ethyl acetate extract of *C. communis* Linn contained effective antibacterial components, and its minimum inhibitory concentrations (MICs) against *Staphylococcus aureus*, *Staphylococcus albus*, *Escherichia coli*, and *Salmonella typhi* were 10 mg/mL. They also identified bioactive compounds n-triacontanol, D-mannitol, p-hydroxycinnamic acid, and daucosteril, the latter two of which showed antibacterial activity and antitussive effect, respectively [4]. However, the underlying molecular mechanisms remain unidentified.

Medicinal plants are a very good source for obtaining a variety of bioactive compounds and drugs [5]. For example, recently, Abed et al. [6] reported the phytochemical composition, and antibacterial, antioxidant, and antidiabetic potential of methanolic extracts of *Cydonia oblonga* bark [6]. Al-Joufi et al. [7] found that *Anabasis articulata* (Forssk.) Moq is a good source of phytochemicals with antibacterial, antioxidant, and antidiabetic potential [7].

To further exploit bioactive nature products in *C. communis* Linn, in this study, we used the methanol and chloroform extraction method, established recently in our research groups [8,9,10], to extract bacteriostatic components from *C. communis* Linn and investigate possible antibacterial mechanisms. The objectives of this study were: (1) to determine inhibition activity of methanol-phase and chloroform-phase crude extracts from *C. communis* Linn against 24 species of common pathogenic bacteria; (2) to purify the methanol-phase extract from *C. communis* Linn using preparative high-performance liquid chromatography (Prep-HPLC) and identify antibacterial compounds using liquid chromatography mass spectrometry (LC-MS); (3) to monitor cell structure changes of four representative bacterial strains mediated by Fragment 1 of the methanol-phase extract; and (4) to decipher molecular mechanisms underlying the antibacterial activity mediated by Fragment 1 by comparative transcriptomic analysis. The results of this study provide useful data for potential pharmaceutical application of *C. communis* Linn against common pathogenic bacteria.

## 2. Results and Discussion

### 2.1. Antibacterial Activity of Methanol- and Chloroform-Phase Crude Extracts from C. communis Linn

Antibacterial substances in fresh leaf and stem tissues of *C. communis* Linn were extracted using the methanol and chloroform extraction method (see the Materials and Methods). We found that the water loss rate of the fresh tissues was 89.57% after freeze-drying treatment of the fresh samples. The observed extraction yields of the methanol-phase and chloroform-phase crude extracts were 30.50% and 16.50%, respectively.

The inhibition rates of the crude extracts against 24 species of common pathogenic bacteria were determined, and the results are shown in Table 1. The chloroform-phase extract from *C. communis* Linn had a bacteriostatic rate of 50.00%, and inhibited two species of Gram-positive bacteria, *S. aureus* and *Enterococcus faecalis*, and 10 species of Gram-negative bacteria: *Enterobacter cloacae*, *E. coli*, *Salmonella paratyphi-A* (ex Kauffmannand Edwards), *Salmonella, Shigella dysenteriae*, *Shigella flexneri*, *Shigella sonnei*, *Vibrio alginolyticus*, *Vibrio parahaemolyticus*, and *Vibrio mimicus* (Table 1).

**Figure 1:**
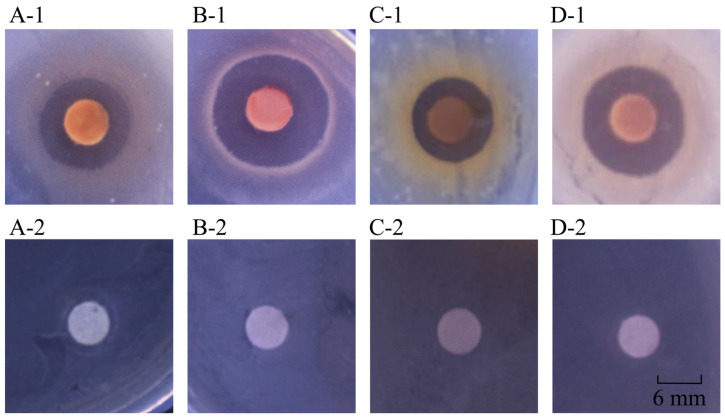
Inhibition activity of the methanol-phase crude extract from C. communis Linn. (**A-1**–**D-1**) *B. cereus* A1-1, *E. faecalis* C1-1, *S. aureus* ATCC25923, and *S. enterica* subsp. *enterica* (ex Kauffmann and Edwards) Le Minor and Popoff serovar Vellore ATCC15611, respectively. (**A-2**–**D-2**) Corresponding negative controls, respectively.

The methanol-phase extract from *C. communis* Linn inhibited the growth of three species of Gram-positive bacteria, *Bacillus cereus*, *S. aureus*, and *E. faecalis*, and 11 species of Gram-negative bacteria: *Aeromonas hydrophila*, *E. cloacae*, *Pseudomonas aeruginosa*, *Salmonella choleraesuis*, *Salmonella enterica* subsp. *enterica* (*ex* Kauffmann and Edwards), *S. dysenteriae*, *S. flexneri*, *V. alginolyticus*, *Vibrio cholerae*, *Vibrio metschnikovii*, and *V. parahaemolyticus* (Table 1, Figure 1).

Methanol and chloroform are organic regents that have been used for effective extraction of bioactive compounds from different pharmacophagous plants [9,10]. The results of this study provided additional evidence of the extraction method for more effectively isolating antibacterial substances from *C. communis* Linn compared to ethyl acetate, with which only four species of bacteria were inhibited: *S. aureus*, *S. albus*, *E. coli*, and *S. typhi* [7].

In this study, the MICs of the two extractions were determined: 32–1024 μg/mL for the methanol-phase extract and 64–1024 μg/mL for the chloroform-phase extract (Table 1). The MIC values of the two extracts were much lower than that of the ethyl acetate extract (10 mg/mL) [7], indicating the higher extraction efficiency of the method used in this study.

Given the higher inhibition rate (58.33%) of the methanol-phase extract from *C. communis* Linn, this crude extract was chosen for further analysis in this study.

### 2.2. Purification of the Methanol-Phase Crude Extract from C. communis Linn

The methanol-phase crude extract from *C. communis* Linn was prepared on a large scale and subjected to Prep-HPLC analysis. This analysis revealed four obviously separated fragments (designated Fragments 1 to 4) by scanning at OD_220_ for 12 min (Figure S1).

These four single fragments were individually collected, and their antibacterial activities were determined (Table 2). The results showed that Fragment 1 highly inhibited the growth of *S. enterica* subsp. *enterica* (*ex* Kauffmann and Edwards) Le Minor and Popoff serovar Vellore ATCC15611, *S. aureus* ATCC25923, *B. cereus* A1-1, and *E. faecalis* C1-1 compared to the control groups (*p <* 0.05). Moreover, the growth of *S. aureus* ATCC8095, *V. alginolyticus* ATCC17749, *V. metschnikovii* ATCC700040, and *V. parahaemolyticus* B5-29 was also significantly repressed (*p* < 0.05). Among these, *S. enterica* subsp. *enterica* (*ex* Kauffmann and Edwards) Le Minor and Popoff serovar Vellore ATCC15611 is a Gram-negative pathogen that can cause mild, self-limiting enterocolitis to systemic (typhoid) diseases in humans [11]. *E. faecalis* is an opportunistic pathogen involved in severe hospital-acquired infections [12]. *S. aureus* can cause pneumonia, endocarditis, and bacteremia upon gaining access to the bloodstream of the host [13]. *B. cereus* can lead to food poisoning and can also cause gastrointestinal disorders and severe systemic infections in humans [14].

In contrast, weak or no antibacterial activity was observed from the other three fragments (Fragments 2 to 4), indicating that the majority of antibacterial compounds existed in Fragment 1 of the methanol-phase extract from *C. communis* Linn.

The MIC values of Fragment 1 were also determined, and the results are presented in Table 2. For example, *S. enterica* ATCC15611 was strongly inhibited by Fragment 1 from *C. communis* Linn. at 128 μg/mL, with *S. aureus* ATCC25923 at 256 μg/mL, and *B. cereus* A1-1 and *E. faecalis* C1-1 at 512 μg/mL, respectively.

### 2.3. Effects of Fragment 1 from C. communis Linn on Bacterial Cell Structure

**Figure 2:**
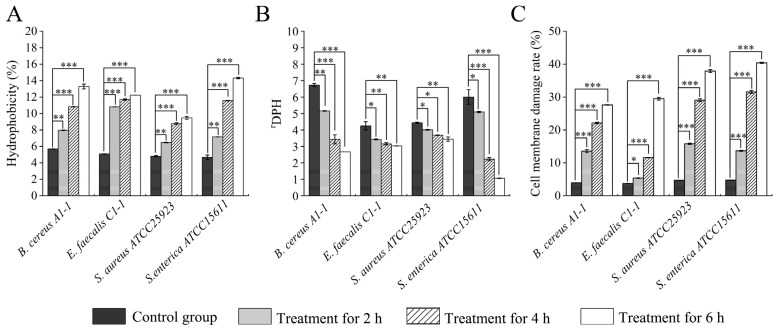
Effects of Fragment 1 from *C. communis* Linn on cell surface hydrophobicity, membrane fluidity and damage of the four bacterial strains. (**A**–**C**) cell surface hydrophobicity, cell membrane fluidity, cell membrane damage, respectively. Results presented as means ± S.D. of three repetitions. *: *p* < 0.05; **: *p* < 0.01; and ***: *p* < 0.001.

Bacterial cell surface hydrophobicity determines the ability of bacteria to adhere to inert surfaces, which plays a key role in bacterial colonization in the host [15]. In this study, after treatment with Fragment 1 from *C. communis* Linn for 2 h, cell surface hydrophobicity of *S. enterica* subsp. *enterica* (*ex* Kauffmann and Edwards) Le Minor and Popoff serovar Vellore ATCC15611, *S. aureus* ATCC25923, *E. faecalis* C1-1 and *B. cereus* A1-1 was significantly increased by 1.53-fold to 2.13-fold compared to the control groups (*p* < 0.05) (Figure 2A). After being treated for 4 h, higher cell surface hydrophobicity was observed (1.89-fold to 2.47-fold). The highest increase (3.06-fold) was observed in the *S. enterica* ATCC15611 treatment group after being treated for 6 h (Figure 2A).

Cell membrane fluidity is also a key property for maintaining the viability of cells and intracellular metabolic functions, particularly under stress [16]. Changes in membrane fluidity have concomitant effects on membrane protein activities and intercellular communication [17]. In this study, the four treatment groups showed significant changes in cell membrane fluidity compared to the control groups (*p* < 0.05) (Figure 2B). For example, after being treated with Fragment 1 from *C. communis* Linn for 2 h, the cell membrane fluidity of *S. enterica* ATCC15611, *S. aureus* ATCC25923, *E. faecalis* C1-1 and *B. cereus* A1-1 was significantly decreased by 2.10-fold, 1.07-fold, 1.04-fold and 1.28-fold, respectively (*p* < 0.05). Upon prolonged treatment for 4 h and 6 h, among the four strains, the most significant decrease in cell membrane fluidity was observed in the *S. enterica* ATCC15611 treatment group (4.80-fold and 5.66-fold) (Figure 2B). The change in membrane lipid composition may contribute to the observed membrane fluidity change by the therapeutic agents [18].

As shown in Figure 2C, cell membrane damage rates were significantly increased in the four treatment groups compared to the control groups (*p* < 0.05). After being treated with Fragment 1 for 2 h, significantly increased damage rates were observed in *B. cereus* A1-1 (3.42-fold), *S. aureus* ATCC25923 (3.36-fold), *S. enterica* ATCC15611 (2.88-fold), and *E. faecalis* C1-1 (1.45-fold) treatment groups (*p* < 0.05). The damage was aggravated after four hours of the treatment, and the most serious damage was observed in *S. enterica* ATCC15611 (6.64-fold), whereas the damage rate of *E. faecalis* C1-1 was the lowest (3.12-fold). The cell membrane damage of *S. enterica* ATCC15611 was also the most severe among the four stains after being treated with Fragment 1 for 6 h (8.52-fold) (Figure 2C).

**Figure 3:**
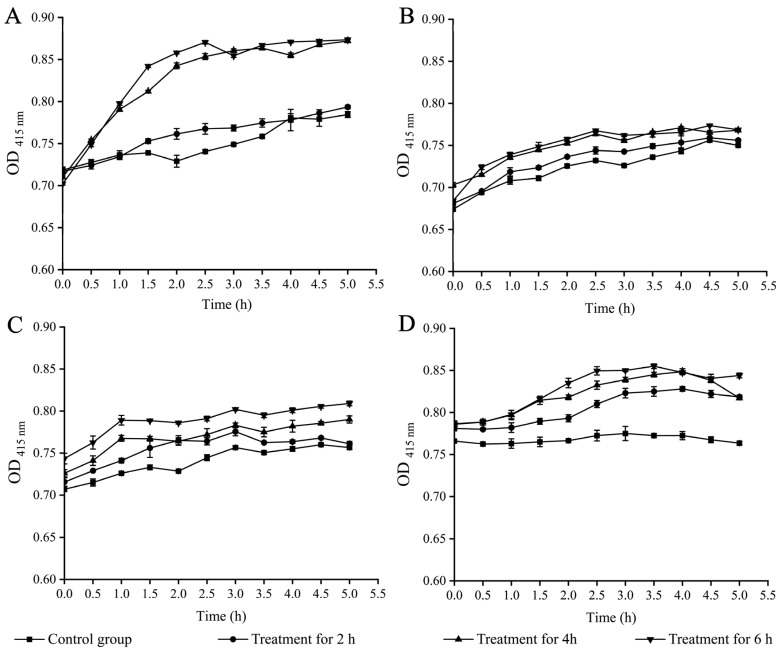
Effects of Fragment 1 from *C. communis* Linn on bacterial inner cell membrane permeability. Results presented as means ± S.D. of three repetitions. (**A**–**D**) *B. cereus* A1-1, *E. faecalis* C1-1, *S. aureus* ATCC25923, and *S. enterica* subsp. *enterica* (*ex* Kauffmann and Edwards) Le Minor and Popoff serovar Vellore ATCC15611, respectively.

Based on the above results, we further examined bacterial inner cell membrane permeability using o-nitrophenyl-*β*-D-galactopyranoside (ONPG) as a probe, and the results are illustrated in Figure 3. For example, the inner cell membrane permeability of *S. enterica* ATCC15611 increased significantly after treatment with Fragment 1 for 2 h (1.07-fold), 4 h (1.08-fold), and 6 h (1.11-fold), respectively (Figure 3A). For *B. cereus* A1-1, its inner cell membrane permeability did not change significantly after the treatment for 2 h (*p* > 0.05), but increased by 1.12-fold and 1.13-fold after the treatment for 4 h, and 6 h, respectively (*p* < 0.05) (Figure 3B). Similarly, *E. faecalis* C1-1 did not show a significant change in inner membrane permeability within 2 h of the treatment, but was significantly increased after 4 h (1.05-fold) and 6 h (1.05-fold) (*p* < 0.05) (Figure 3C). *S. aureus* ATCC25923 had no significant change in inner cell membrane permeability after being treated for 2 h and 4 h, but showed significantly increased permeability after treatment for 6 h (1.08-fold) (Figure 3D). These results indicated that Fragment 1 from *C. communis* Linn displays different damage effects on inner cell membrane permeability of the four bacterial strains.

**Figure 4:**
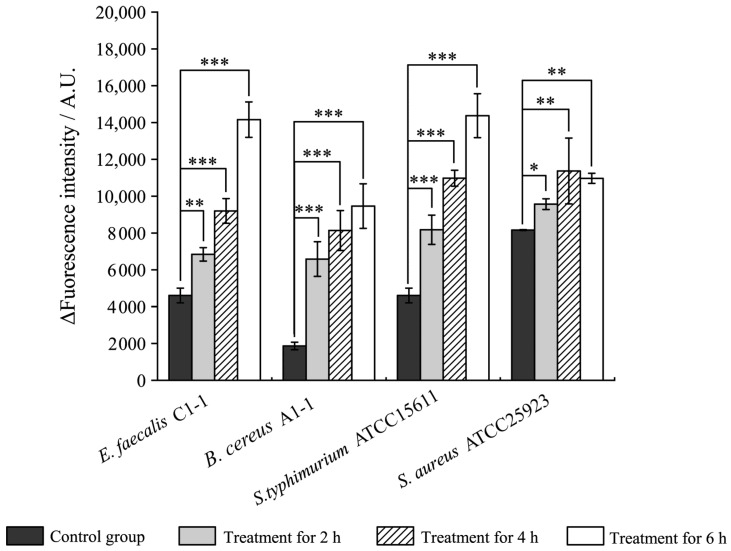
Effects of Fragment 1 from *C. communis* Linn on bacterial outer cell membrane permeability. Results presented as means ± S.D. of three repetitions. *: *p* < 0.05; **: *p* < 0.01; and ***: *p* < 0.001.

We also examined bacterial outer membrane permeability using N-Phenyl-1-naphthylamine (NPN) as a probe, and the results are presented in Figure 4. The outer membrane permeability in the four treatment groups was all significantly increased (1.17-fold to 3.52-fold) after the treatment with Fragment 1 for 2 h (*p* < 0.05). A higher increase in outer membrane permeability was observed in *B. cereus* A1-1 (4.36-fold) and *S. enterica* ATCC15611 (2.38-fold) groups after being treated for 4 h (*p* < 0.001), followed by *E. faecalis* C1-1 (2.00-fold), and *S. aureus* ATCC25923 (1.39-fold) (*p* < 0.01). After 6 h of the treatment, the outer membrane permeability of *B. cereus* A1-1 also increased most significantly (5.07-fold), while that of *S. aureus* ATCC25923 was the opposite (1.34-fold).

In conclusion, these results indicated that Fragment 1 from *C. communis* Linn can affect cell membrane structures of *S. enterica* ATCC15611, *E. faecalis* C1-1, *S. aureus* ATCC25923 and *B. cereus* A1-1, particularly increasing cell surface hydrophobicity and membrane permeability, but reducing membrane fluidity, and significantly damage cell membrane structures, in agreement with bacterial surface structure changes observed by scanning electron microscopy (SEM) analysis (see below). The damage to cell membranes was conducive to the penetration of fragment 1 of *C. communis* Linn into bacterial cells, consequently influencing intracellular metabolisms and thereby inhibiting bacterial growth and even leading to bacterial death.

### 2.4. Bacterial Cell Surface Structure Changes Observed by the SEM Assay

**Figure 5:**
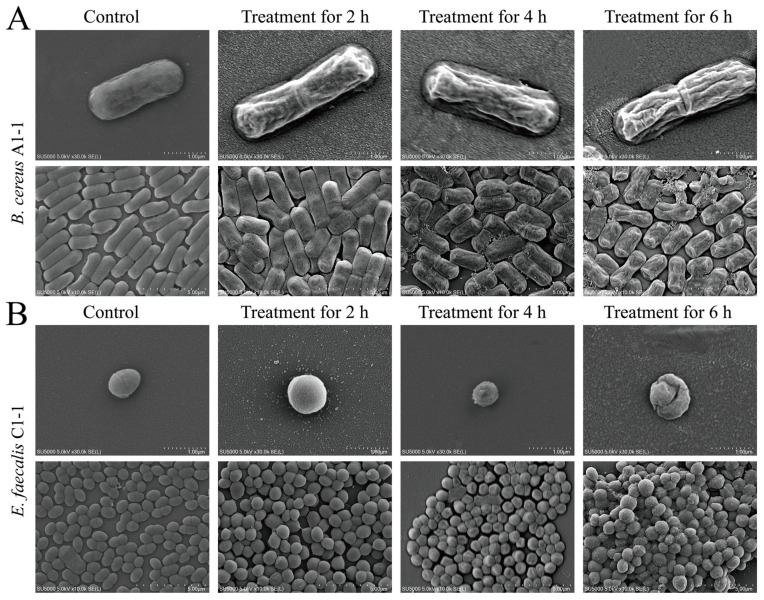

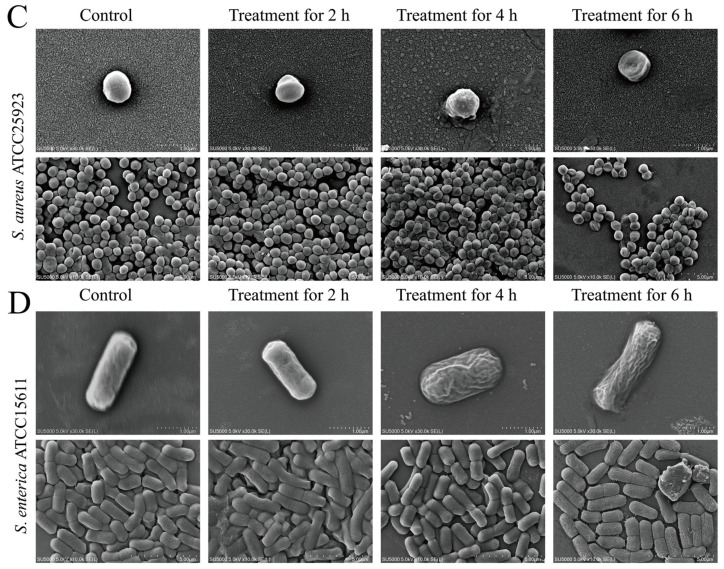
SEM observation of cell surface structure of the four bacterial strains treated with Fragment 1 for different times. (**A**–**D**) *B. cereus* A1-1, *S. aureus* ATCC25923, *E. faecalis* C1-1, and *S. enterica* subsp. *enterica* (*ex* Kauffmann and Edwards) Le Minor and Popoff serovar Vellore ATCC15611, respectively.

Based on the above results, we further observed bacterial cell surface structure changes by the SEM assay (Figure 5). In the control groups, cells of coccus were spherical or slightly elliptical with smooth surface and clear structure, while cells of bacillus were rods, round at both ends, smooth and complete in surface. For example, *B. cereus* A1-1 was a rod-shaped Gram-positive bacterium. The bacterial cells in the control group were intact in shape with visible pili. However, after the treatment with Fragment 1 from *C. communis* Linn for 2 h, the cell surface shrank. The change showed a treatment time-dependent mode, and a large number of the bacterial cells broke, and cellular contents leaked after the treatment for 6 h.

Similarly, for the Gram-positive *S. aureus* ATCC25923, in the control group, the surface of the bacterial cells was clear, smooth and wrinkle-free with the typical spherical structure. However, after being treated with Fragment 1 for 2 h, although most cells were plump and spherical with complete cell structure, a small number of the cells shrank on the surface. After treatment for 4 h, the surface of most cells showed slight wrinkles, and even collapsed and lost the spherical structure after the treatment for 6 h.

For the Gram-positive *E. faecalis* C1-1, the control group cells were single, paired or short chains of ovoid cocci with smooth surface and intact morphological structure. No obvious change was observed after treatment with Fragment 1 for 2 h. However, after being treated for 4 h, a large number of the bacterial cells had sunk on the surface, and the cells severely shrank and deformed. After the treatment for 6 h, a large amount of cellular content leaked and the bacterial cells lost the spherical structure.

For the Gram-negative *S. enterica* subsp. *enterica* (*ex* Kauffmann and Edwards) Le Minor and Popoff serovar Vellore ATCC15611, the control group was morphologically intact with a short rod shape. It did not change significantly after treatment with Fragment 1 for 2 h. However, after being treated for 4 h, the cell surface slightly contracted, and some cells broke. The bacterial cells shrank severely after treatment for 6 h.

These results demonstrated that Fragment 1 from *C. communis* Linn (1 MIC) can cause varying degrees of damage on cellular surface structure of both Gram-positive and Gram-negative bacteria, especially the coccus, which showed the most obvious shrinkage, fracture or cavities on the cell surface.

### 2.5. Identification of Potential Antibacterial Compounds in Fragment 1 from C. communis Linn

In order to identify antibacterial compounds in *C. communis* Linn, Fragment 1 was further subjected to UHPLC-MS analysis. Approximately 65 compounds with known functions were identified, the highest percentage of which was quercetin-3-o-glucuronide (19.35%), followed by glutamine (8.69%), sucrose (6.46%), methyl gallate (4.93%), and indole (4.52%) (Table 3). The major compound classes included flavonoids, alkaloids, phenols, terpenoids, and steroids. Studies have indicated that the flavonoid quercetin-3-o-glucuronide has anti-inflammatory, antiviral, and antiallergic properties [19,20]. Quercetin-3-o-glucuronide is a pharmacologically active flavonol glucuronide, and Kawai recently found unique actions at sites of inflammation, including specific accumulation in macrophages and the following deconjugation into active aglycone, catalyzed by the macrophage-derived β-glucuronidase [21].

Alkaloids exhibit various biological functions such as antitumor, antiviral, antimicrobial and anti-inflammatory activities [22]. Terpenoids, such as kaurenoic acid, miltirone, kirenol, and shionone, identified in this study, are the largest class of natural products, most of which are derived from plants [23]. They play important roles in food and pharmaceutical fields due to diverse biological and pharmacological activities [24]. Terpenoids have huge potential against microorganisms through different mechanisms, such as membrane disruption, anti-quorum sensing, and inhibition of protein synthesis [25]. The combination therapy of terpenoids and antimicrobial agents has increased the potency of treatment against multidrug-resistant microorganisms by showing synergism to each other [25]. For example, methyl gallate, identified in this study, can inhibit bacterial virulence and reduce membrane integrity and cell survival rate of *S. typhimurium* [26]. Additionally, trigonelline, identified in this study, has antibacterial, antiviral and antitumor activities, with therapeutic potential for diabetes and central nervous system disease [27].

### 2.6. Differential Transcriptomes Mediated by Fragment 1 from C. communis Linn

To obtain further insights into the genome-wide gene expression changes mediated by Fragment 1 from *C. communis* Linn, we determined transcriptomes of *S. enterica* subsp. *enterica* (*ex* Kauffmann and Edwards) Le Minor and Popoff serovar Vellore ATCC15611, *S. aureus* ATCC25923, *B. cereus* A1-1, and *E. faecalis* C1-1 treated with Fragment 1 (1 MIC) for 6 h using Illumina RNA sequencing. The complete lists of differently expressed genes (DEGs) in the four strains are available in the NCBI SRA database (https://submit.ncbi.nlm.nih.gov/subs/bioproject/, accessed on 13 October 2022) under the accession number PRJNA890025.

#### 2.6.1. Major Altered Metabolic Pathways in *S. enterica* subsp. *enterica* (*ex* Kauffmann and Edwards) Le Minor and Popoff Serovar Vellore ATCC15611 Mediated by Fragment 1 from *C. communis* Linn

**Figure 6:**
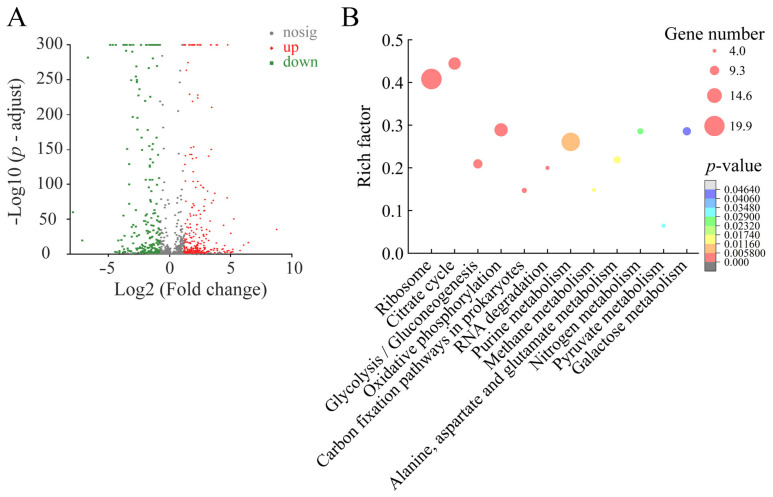
The 12 significantly altered metabolic pathways in *S. enterica* subsp. *enterica* (*ex* Kauffmann and Edwards) Le Minor and Popoff serovar Vellore ATCC15611 mediated by Fragment 1 from *C. communis* Linn. (**A**,**B**) Volcano plot of differential gene expression and significantly changed metabolic pathways in *S. enterica* subsp. *enterica* (*ex* Kauffmann and Edwards) Le Minor and Popoff serovar Vellore ATCC15611, respectively.

Approximately 11.05% (506/4578) of *S. enterica* subsp. *enterica* (*ex* Kauffmann and Edwards) Le Minor and Popoff serovar Vellore ATCC15611 genes were expressed differently in the treatment group compared to the control group. Among these, 246 DEGs showed higher transcription levels (FC ≥ 2.0), whereas 260 DEGs were downregulated (FC ≤ 0.5) (*p* < 0.05). Approximately 12 significantly changed metabolic pathways were identified, including the ribosome; citrate cycle; glycolysis/gluconeogenesis; oxidative phosphorylation; carbon fixation pathways in prokaryotes; RNA degradation; purine metabolism; methane metabolism; alanine, aspartate and glutamate metabolism; nitrogen metabolism; pyruvate metabolism; and galactose metabolism (Figure 6, Table 4).

Of note, approximately 68 DEGs involved in 12 changed metabolic pathways were significantly downregulated at the transcription level in *S. enterica* subsp. *enterica* (*ex* Kauffmann and Edwards) Le Minor and Popoff serovar Vellore ATCC15611 (0.009-fold to 0.500-fold) (*p* < 0.05) (Table 4). For example, in the citrate cycle, the expression of 10 DEGs was significantly inhibited (0.084-fold to 0.500-fold) (*p* < 0.05). Remarkably, the DEGs encoding a fumarate hydratase (FH) class I aerobic (*SPC_2263*) and a succinate dehydrogenase catalytic subunit (*SPC_0731*) were greatly downregulated (0.084-fold and 0.097-fold). The FH catalyzes the conversion of fumarate to L-malate as part of the tricarboxylic acid cycle (TCA) [28]. Fumarate has been shown to inhibit α-ketoglutarate-dependent dioxygenases that are involved in DNA and histone demethylation [29]. In this study, five DEGs encoding key enzymes in the oxidative phosphorylation were also significantly repressed (0.057-fold to 0.497-fold) (*p* < 0.05), including a succinate dehydrogenase cytochrome b556 small membrane subunit (*SPC_0730*), a NADH dehydrogenase alpha subunit (*SPC_1381*), a NADH dehydrogenase I chain 2CD (*SPC_1383*), and a NADH dehydrogenase subunit H (*SPC_1387*), and an inorganic pyrophosphatase (*SPC_4565*). Additionally, in the carbon fixation pathways in prokaryotes, the DGEs encoding an acetyl-coenzyme synthetase (*SPC_4339*) and a succinate dehydrogenase catalytic subunit (*SPC_0732*) were significantly downregulated as well (0.047-fold and 0.127-fold) (*p* < 0.05). Succinate dehydrogenase is a heterotetrameric protein complex that links the TCA with the electron transport chain [30]. In the methane metabolism, the expression of three DEGs was also significantly repressed (0.225-fold to 0.434-fold) (*p* < 0.05), including a phosphate acetyltransferase (*SPC_1369*), a phosphoenolpyruvate synthase (*SPC_2381*), and a formate dehydrogenase-O gamma subunit (*SPC_4138*). The above five metabolic pathways were involved in energy metabolism, the significantly downregulation of which indicated that the energy supply is deficient in *S. enterica* ATCC15611 mediated by Fragment 1 from *C. communis* Linn.

Three metabolic pathways related to the carbohydrate metabolism were also downregulated significantly (*p* < 0.05). For example, in glycolysis/gluconeogenesis, the expression of three DEGs was significantly repressed (0.060-fold to 0.417-fold) (*p* < 0.05), including an aldehyde dehydrogenase B (*SPC_3760*), a fructose-1-bisphosphatase (*SPC_4566*), and a glucose-6-phosphate isomerase (*SPC_4282*). In the galactose metabolism, the expression of five DEGs was also significantly downregulated at the transcription level (0.009-fold to 0.397-fold) (*p* < 0.05), including an alpha-galactosidase (*SPC_4361*), a galactose-1-phosphate uridylyltransferase (*SPC_0771*), an UDP-galactose 4-epimerase (*SPC_0772*), a galactonate dehydratase (*SPC_3914*), and a 2-oxo-3-deoxygalactonate kinase (*SPC_3916*). Among these, notably, the expression of alpha-galactosidase (*SPC_4361*) was strongly inhibited (0.009-fold) (*p* < 0.05), which is an exoglycosidase that targets galactooligosaccharides such as raffinose, melibiose and branched polysaccharides and catalyzes the hydrolysis of α-1,6 linked terminal galactose residues [31]. Alpha galactosidase is also directly or indirectly involved in the synthesis of extracellular polysaccharides, which are major elements of the intercellular matrix [32]. In this study, the greatly inhibited alpha-galactosidase in *S. enterica* ATCC15611 was consistent with the impaired cell surface structure observed by the SEM analysis.

In bacteria, ribosome can bind to nascent RNA emerging from the transcribing RNA polymerase and initiate protein translation, which plays critical roles in cellular physiology [33]. Bacterial ribosome comprises two subunits, denoted 30S and 50S [34]. In this study, comparative transcriptome analysis revealed 19 significantly downregulated DEGs related to ribosome in *S. enterica* ATCC15611 (0.064-fold to 0.484-fold) (*p* < 0.05), e.g., 30S ribosomal protein S10 (*SPC_3510*), 50S ribosomal protein L2 (*SPC_3506*), 50S ribosomal protein L4 (*SPC_3508*), and 50S ribosomal protein L3 (*SPC_3509*), indicating that protein translation was significantly inhibited in *S. enterica* ATCC15611 after being treated with Fragment 1.

Conversely, in nitrogen metabolism, four DEGs were significantly upregulated (3.204-fold to 29.218-fold) (*p* < 0.05): a respiratory nitrate reductase 2 beta chain (*SPC_2160*), a respiratory nitrate reductase 2 alpha chain (*SPC_2161*), a nitrite reductase large subunit (*SPC_3544*), and a hydroxylamine reductase (*SPC_0939*). Notably, the expression of hydroxylamine reductase was strongly upregulated (29.218-fold), which catalyzes the reduction of hydroxylamine to ammonia to generate NAD^+^ and H_2_O in the presence of NADH. It has been suggested that hydroxylamine reductase might play some role in nitrogen respiration [35]. Similarly, in the purine metabolic pathway, the expression of 10 DEGs was significantly upregulated (2.018-fold to 23.21-fold) (*p* < 0.05), e.g., a nucleoside diphosphate kinase (*SPC_1128*), a guanylate kinase (*SPC_3822*), an adenylosuccinate synthetase (*SPC_4513*), an anaerobic ribonucleoside triphosphate reductase (*SPC_4584*), a nucleotidase (*SPC_4695*), and a phosphopentomutase (*SPC_4705*). For instance, adenylosuccinate synthetase (*SPC_4513*) is involved in the synthesis of adenosine monophosphate [36]. Interestingly, anaerobic ribonucleoside triphosphate reductase (*SPC_4584*) was highly upregulated (23.21-fold), which is an iron–sulfur protein carrying an oxygen-sensitive organic radical and is essential for catalyzing the reduction of nucleoside triphosphate [37].

The comparative transcriptomic data demonstrated that Fragment 1 from *C. communis* Linn can significantly alter 12 metabolic pathways in *S. enterica* subsp. *enterica* (*ex* Kauffmann and Edwards) Le Minor and Popoff serovar Vellore ATCC15611, which results in inhibited carbohydrate metabolism, insufficient energy supply, and downregulated protein translation, and consequently affects bacterial survival.

#### 2.6.2. Major Altered Metabolic Pathways in *S. aureus* ATCC25923 Mediated by Fragment 1 from *C. communis* Linn

**Figure 7:**
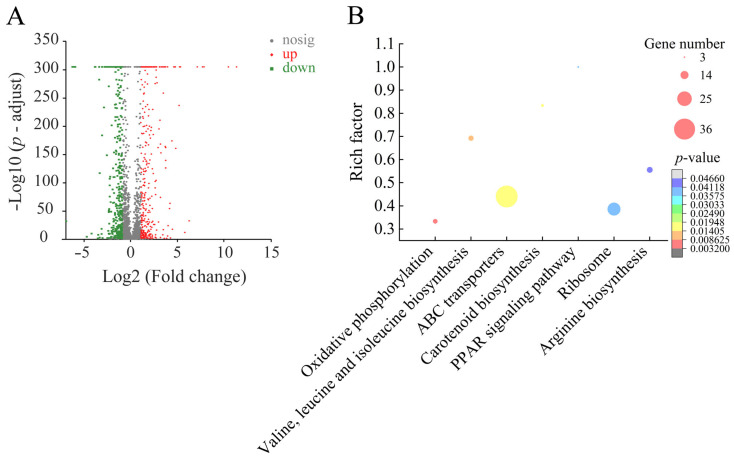
The seven significantly altered metabolic pathways in *S. aureus* ATCC25923 mediated by Fragment 1 from *C. communis* Linn. (**A**,**B**) Volcano plot of differential gene expression and significantly changed metabolic pathways in *S. aureus* ATCC25923, respectively.

Approximately 27.77% (803/2892) of *S. aureus* ATCC25923 genes were expressed differently in the treatment group compared to the control group. Among the identified DEGs, 310 showed higher transcription levels (FC ≥ 2.0), whereas 493 were significantly downregulated (FC ≤ 0.5). Comparative transcriptome analyses revealed seven significantly changed metabolic pathways in *S. aureus* ATCC25923: oxidative phosphorylation, valine, leucine, and isoleucine biosynthesis, ATP-binding cassette (ABC) transporters, carotenoid biosynthesis, peroxisome proliferator-activated receptor signaling pathway, ribosome, and arginine biosynthesis (Figure 7, Table 5).

Notably, approximately 71 DEGs involved in the seven changed metabolic pathways were significantly downregulated (0.012-fold to 0.494-fold) (*p* < 0.05) (Table 5). For example, in oxidative phosphorylation, the expression of five DEGs was significantly inhibited at the transcription level (0.249-fold to 0.350-fold) (*p* < 0.05), including an ATP synthase F1 epsilon subunit (*SAOUHSC_02340*), a quinol oxidase subunit IV putative (*SAOUHSC_00999*), a cytochrome c oxidase subunit III putative (*SAOUHSC_01000*), a quinol oxidase subunit I (*SAOUHSC_01001*), and a quinol oxidase AA3 subunit II putative (*SAOUHSC_01002*). This metabolic pathway was related to energy metabolism, the downregulation of which indicated insufficient energy supply in *S. aureus* ATCC25923.

In valine, leucine, and isoleucine biosynthesis, nine DEGs were also significantly downregulated (0.132-fold to 0.331-fold) (*p* < 0.05), including a dihydroxy-acid dehydratase (*SAOUHSC_02281*), an acetolactate synthase large subunit biosynthetic type (*SAOUHSC_02282*), a conserved hypothetical protein (*SAOUHSC_02283*), a ketol-acid reductoisomerase (*SAOUHSC_02284*), a 2-isopropylmalate synthase (*SAOUHSC_02285*), a 3-isopropylmalate dehydrogenase (*SAOUHSC_02286*), a 3-isopropylmalate dehydratase large subunit (*SAOUHSC_02287*), a 3-isopropylmalate dehydratase small subunit (*SAOUHSC_02288*), and a putative threonine dehydratase (*SAOUHSC_02289*). For instance, 2-isopropylmalate synthase (*SAOUHSC_02285*, 0.141-fold) catalyzes the first step of leucine biosynthesis and is regulated via feedback inhibition by leucine [38], while 3-isopropylmalate dehydrogenase (*SAOUHSC_02286*, 0.142-fold) catalyzes the irreversible oxidative decarboxylation of 3-isopropylmalate to 2-ketoisocaproate that is finally converted into leucine by a branched-chain aminotransferase [39]. Similarly, in arginine biosynthesis, five DEGs were also significantly downregulated (0.246-fold to 0.397-fold) (*p* < 0.05), including an acetylglutamate kinase putative (*SAOUHSC_00147*), a glutamate N-acetyltransferase/amino-acid acetyltransferase (*SAOUHSC_00148*), an ornithine carbamoyltransferase (*SAOUHSC_01128*), a carbamate kinase (*SAOUHSC_01129*), and a nitric oxide synthase oxygenase domain putative (*SAOUHSC_02134*). Amino acid metabolism of bacteria is related to energy supply [40]. These downregulated pathways indicated that Fragment 1 can also affect the energy supply in *S. aureus* ATCC25923.

Comparative transcriptomic analysis also revealed that 24 DEGs encoding ABC transporters were significantly downregulated in *S. aureus* ATCC25923 (0.012-fold to 0.483-fold) (*p* < 0.05). ABC transporters are also known as efflux pumps that mediate the cross-membrane transportation of various endo- and xenobiotic molecules energized by ATP hydrolysis [41]. Therefore, ABC transporters have been considered closely in multidrug resistance (MDR), where the efflux of structurally distinct chemotherapeutic drugs causes reduced therapeutic efficacy [42]. In this study, the downregulated expression of these DEGs indicated that Fragment 1 from *C. communis* Linn can repress the pumping out of harmful substances by *S. aureus* ATCC25923 in order to eliminate cell damage.

Approximately 21 DEGs related to ribosome were also significantly downregulated in *S. aureus* ATCC25923 (0.258-fold to 0.494-fold) (*p* < 0.05). Likewise, in the peroxisome proliferator-activated receptor signaling pathway, the expression of two DEGs was significantly repressed, including a glycerol kinase (*SAOUHSC_01276*) (0.337-fold) and a conserved hypothetical protein (*SAOUHSC_00198*) (0.492-fold). In addition, in carotenoid biosynthesis, the expression of three DEGs was significantly inhibited (0.222-fold to 0.324-fold) (*p* < 0.05), including a squalene synthase putative (*SAOUHSC_02877*), a squalene desaturase putative (*SAOUHSC_02879*), and a conserved hypothetical protein (*SAOUHSC_02880*).

Comparative transcriptomic analysis revealed that Fragment 1 from *C. communis* Linn can significantly change seven metabolic pathways in *S. aureus* ATCC25923 and inhibit oxidative phosphorylation, valine, leucine, and isoleucine biosynthesis, ABC transporters, carotenoid biosynthesis, peroxisome proliferator-activated receptor signaling pathway, ribosome, and arginine biosynthesis, which consequently may result in reduced ABC transport, energy metabolism, signal transduction, and protein synthesis.

#### 2.6.3. Major Altered Metabolic Pathways in *B. cereus* A1-1 Mediated by Fragment 1 from *C. communis* Linn

**Figure 8:**
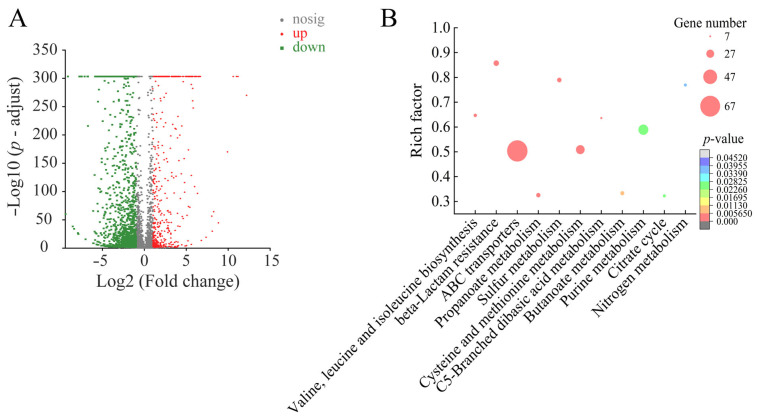
The 11 significantly altered metabolic pathways in *B. cereus* A1-1 mediated by Fragment 1 from *C. communis* Linn. (**A**,**B**) Volcano plot of differential gene expression and significantly changed metabolic pathways in the bacterium.

Approximately 35.66% (1931/5415) of *B. cereus* A1-1 genes were expressed significantly in the treatment group compared to the control group. Among these, 437 DEGs showed higher transcription levels (FC ≥ 2.0), whereas 1494 DEGs were downregulated (FC ≤ 0.5). Comparative transcriptome analysis revealed 11 significantly changed metabolic pathways in *B. cereus* A1-1: valine, leucine, and isoleucine biosynthesis, beta-lactam resistance, ABC transporters, propanoate metabolism, sulfur metabolism, cysteine and methionine metabolism, C5-branched dibasic acid metabolism, butanoate metabolism, purine metabolism, citrate cycle, and nitrogen metabolism (Table 6, Figure 8).

Approximately 181 DEGs involved in 11 changed metabolic pathways in *B. cereus* A1-1 were significantly downregulated at the transcription level (0.003-fold to 0.495-fold) (*p* < 0.05) (Table 6). Notably, two pathways related to amino acid metabolism were significantly inhibited (0.005-fold to 0.493-fold) (*p* < 0.05), including valine, leucine, and isoleucine biosynthesis and cysteine and methionine metabolism. For instance, the DEG encoding a branched-chain amino acid aminotransferase (*BCN_1374*) in valine, leucine, and isoleucine biosynthesis was significantly downregulated (0.158-fold) (*p* < 0.05), which acts upon branched-chain amino acids to regulate sulfur amino acid metabolism [43]. Two DEGs encoding homoserine dehydrogenase (HSD, *BCN_1887* and *BCN_5298*) in cysteine and methionine metabolism were strongly repressed (0.005-fold and 0.210-fold) (*p* < 0.05), which catalyzes NAD(P)H-dependent production of homoserine from L-aspartate-4-semialdehyde and functions in the aspartate pathway, catalyzing the synthesis of cysteine, threonine, and methionine from aspartate in plants, fungi, yeast, bacteria, and archaea [44].

Three pathways related to energy metabolism were also significantly repressed (0.017-fold to 0.379-fold) (*p* < 0.05), including sulfur metabolism, citrate cycle, and nitrogen metabolism. Additionally, three pathways involved in carbohydrate metabolism were significantly downregulated a (0.003-fold to 0.457-fold) (*p* < 0.05), including propanoate metabolism, C5-branched dibasic acid metabolism, and butanoate metabolism.

Interestingly, approximately 18 DEGs encoding beta-lactam resistance components were also significantly downregulated in *B. cereus* A1-1 (0.050-fold to 0.491-fold) (*p* < 0.05), including oligopeptide ABC transporter oligopeptide-binding proteins (*BCN_1158*, *BCN_0187*, *BCN_1796* and *BCN_3396*), oligopeptide ABC transporter oligopeptide-binding proteins putative (*BCN_0190*, *BCN_1956*, *BCN_2708* and *BCN_3395*), an oligopeptide-binding protein (OppA, *BCN_1762*), penicillin-binding proteins (*BCN_1046*, *BCN_1433*, *BCN_1533* and *BCN_2267*), oligopeptide ABC transporter permease proteins (*BCN_1159* and *BCN_1160*), an oligopeptide ABC transporter ATP-binding protein (*BCN_1161* and *BCN_1162*), and a beta-lactamase (*BCN_2412*). The beta-lactam resistance pathway has been suggested to be involved in antibiotic resistance against β-lactams [45]. For instance, remarkably, the expression of the OppA (*BCN_1762*) was highly repressed (0.069-fold), which plays a crucial role in nutrient uptake and sensing the external environment in bacteria [46]. It has been reported that OppA also functions in the uptake of aminoglycoside antibiotics in *E. coli* K-12 [47].

In purine metabolism, the expression of 15 DEGs was significantly inhibited (0.073-fold to 0.495-fold) (*p* < 0.05), e.g., an inosine–uridine-preferring nucleoside hydrolase family protein (*BCN_2749*), a deoxynucleoside kinase family protein (BCN_0015), an inosine-5′-monophosphate dehydrogenase (*BCN_0009*), a 3′-cyclic-nucleotide 2′-phosphodiesterase (*BCN_4039*), and a deoxynucleoside kinase family protein (*BCN_0016*). Conversely, 18 DEGs in purine metabolism were significantly upregulated (2.023-fold to 15.341-fold) (*p* < 0.05), e.g., a phosphoribosylamine-glycine ligase (*BCN_0296*), a xanthine phosphoribosyltransferase (*BCN_1550*), a phosphoribosylaminoimidazole-succinocarboxamide synthase (*BCN_0287*), a phosphoribosylglycinamide formyltransferase (*BCN_0293*), a pyruvate kinase (*BCN_4500*), and an adenylate kinase (*BCN_0131*). Of these, the pyruvate kinase *BCN_4500* catalyzes the conversion of phosphoenolpyruvate and ADP to pyruvate and ATP in glycolysis and plays a very important role in regulating cell metabolism [48]. Purines are structural components of some coenzymes and also contribute to modulate energy metabolism and signal transduction [49]. Purine metabolism maintains cellular pools of adenylate and guanylate via synthesis and degradation of purine nucleotides [50].

Similarly to *S. aureus* ATCC25923, the expression of 51 DEGs in ABC transporters was significantly downregulated in *B. cereus* A1-1 (0.022-fold to 0.438-fold) (*p* < 0.05). Notably, the DEG encoding a cobalt transport protein (*BCN_2523*) was highly downregulated (0.030-fold), which belongs to a family of secondary metal transporters in prokaryotes and fungi, characterized by an eight-transmembrane-domain architecture and mediating high-affinity uptake of cobalt and/or nickel ions into cells [51].

In contrast, like *S. enterica* ATCC15611, eight DEGs involved in nitrogen metabolism were significantly upregulated in *B. cereus* A1-1 (2.155-fold to 8.415-fold) (*p* < 0.05), including a nitrate transporter (*BCN_2097*), a glutamate synthase large subunit putative (*BCN_0507*), a carbonic anhydrase prokaryotic type putative (*BCN_4708*), a respiratory nitrate reductase alpha subunit (*BCN_2086*), a respiratory nitrate reductase beta subunit (*BCN_2087*), a respiratory nitrate reductase gamma subunit (*BCN_2089*), a nitrite reductase [NAD(P)H] large subunit (*BCN_2105*), and a nitrite reductase [NAD(P)H] small subunit (*BCN_2104*).

Comparative transcriptomic analysis indicated that Fragment 1 from *C. communis* Linn. can increase nitrogen metabolism and purine metabolism in *B. cereus* A1-1, but inhibits ABC transporters, valine, leucine, and isoleucine biosynthesis, cysteine and methionine metabolism, propanoate metabolism, sulfur metabolism, C5-branched dibasic acid metabolism, butanoate metabolism, citrate cycle, and beta-lactam resistance, which likely results in repressed energy metabolism, transport system, carbohydrate metabolism, protein synthesis, and drug resistance, and consequently influences cell growth and proliferation of *B. cereus* A1-1.

#### 2.6.4. Major Altered Metabolic Pathways in *E. faecalis* C1-1 Mediated by Fragment 1 from *C. communis* Linn

**Figure 9:**
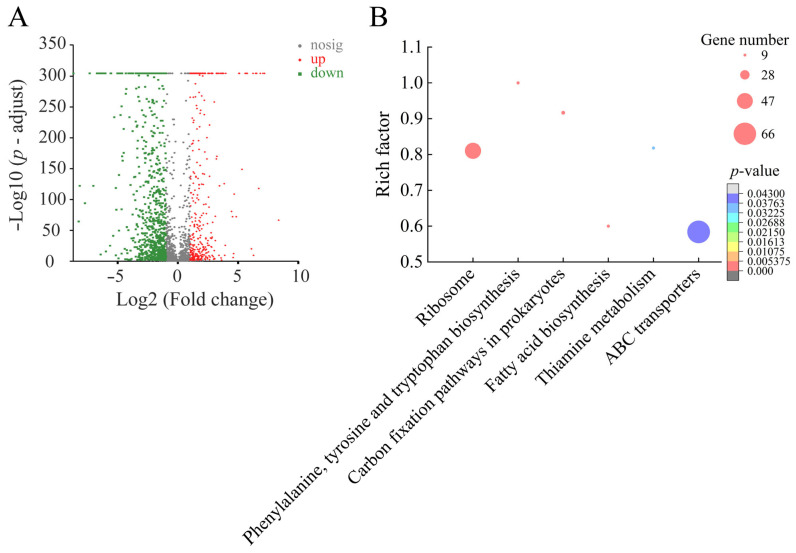
The six significantly altered metabolic pathways in *E. faecalis* C1-1 mediated by Fragment 1 from *C. communis* Linn. (**A**,**B**) Volcano plot of differential gene expression and significantly changed metabolic pathways in *E. faecalis* C1-1.

Approximately 44.96% (1204/2678) of *E. faecalis* C1-1 genes were expressed differently in the treatment group compared to the control group. Among these, 353 DGEs showed higher transcription levels (FC ≥ 2.0), whereas 851 DEGs were downregulated (FC ≤ 0.5). The comparative transcriptomic analysis revealed six significantly changed metabolic pathways in *E. faecalis* C1-1: the ribosome; phenylalanine, tyrosine, and tryptophan biosynthesis; carbon fixation pathways in prokaryotes; fatty acid biosynthesis; thiamine metabolism; and ABC transporters (Figure 9, Table 7).

The expression of a large number of DEGs (*n* = 52) encoding ABC transporters was significantly depressed in *E. faecalis* C1-1 (0.002-fold to 0.472-fold) (*p* < 0.05). Notably, the DEG encoding the substrate-binding protein (SBP) (*IUJ47_RS12795*) was extremely downregulated (0.002-fold), which is a key determinant of substrate specificity and high affinity of ABC uptake systems in bacteria and archaea. Most prokaryotes have many SBP-dependent ABC transporters that recognize a broad range of ligands from metal ions to amino acids, sugars and peptides [52].

Approximately nine DEGs in the phenylalanine, tyrosine and tryptophan biosynthesis were also significantly downregulated in *E. faecalis* C1-1 (0.021-fold to 0.304-fold): a shikimate dehydrogenase (*IUJ47_RS10435*), a 3-deoxy-7-phosphoheptulonate synthase (*IUJ47_RS10440*), a type I 3-dehydroquinate dehydratase (*IUJ47_RS11275*), a 3-dehydroquinate synthase (*IUJ47_RS10445*), a prephenate dehydrogenase (*IUJ47_RS10455*), a 3-phosphoshikimate 1-carboxyvinyltransferase (*IUJ47_RS10460*), a shikimate kinase (*IUJ47_RS10465*), a chorismate synthase (*IUJ47_RS10450*), and a prephenate dehydratase (*IUJ47_RS10470*).

Notably, nine DEGs in fatty acid biosynthesis were significantly inhibited in *E. faecalis* C1-1 as well (0.075-fold to 0.344-fold) (*p* < 0.05), e.g., a ketoacyl-ACP synthase III (KAS III, *IUJ47_RS01560*), a 3-hydroxyacyl-ACP dehydratase FabZ (*IUJ47_RS01525*), an enoyl-ACP reductase FabI (*IUJ47_RS05020*), and a beta-ketoacyl-ACP synthase II (*IUJ47_RS05025*). The fatty acid biosynthesis is an essential cellular process that converts nutrients into metabolic intermediates for membrane biosynthesis, energy storage and the generation of signal molecules [53]. In this study, the expression of the KAS III (*IUJ47_RS01560*) was strongly downregulated (0.075-fold) in fatty acid biosynthesis, which catalyzes a carbon–carbon bond-formation reaction that involves the α-carbon of a thioester and the carbonyl carbon of another thioester, the formation of acetoacetyl-CoA in mevalonate biosynthesis, tailoring processes via C-O bond formation or esterification, as well as amide formation [54].

Additionally, nine DEGs in thiamine metabolism were all significantly downregulated (0.161-fold to 0.421-fold) (*p* < 0.05), including a thiaminase II (*IUJ47_RS01260*), a hydroxyethylthiazole kinase (*IUJ47_RS01310*), an alkaline phosphatase (*IUJ47_RS02170*), a bifunctional hydroxymethylpyrimidine kinase/phosphomethylpyrimidine kinase (*IUJ47_RS01300*), a tRNA 4-thiouridine (8) synthase ThiI (*IUJ47_RS02080*), a adenylate kinase (IUJ47_RS04630) a cysteine desulfurase (*IUJ47_RS05190*), a thiamine phosphate synthase (*IUJ47_RS01305*), and a ribosome small subunit-dependent GTPase (*AIUJ47_RS02855*). Thiamine is a crucial molecule in all living organisms. The physiologically active form of thiamine, thiamine pyrophosphate (TPP), plays an important role as a cofactor in various essential cellular processes, including carbohydrate, lipid, and amino acid metabolism [55].

As with *S. enterica* ATCC15611 and *S. aureus* ATCC25923, the expression of many DEGs (n = 42) involved in the ribosome was significantly downregulated in *E. faecalis* C1-1 (0.021-fold to 0.471-fold) (*p* < 0.05). The expression of 10 DEGs involved in carbon fixation pathways in prokaryotes was also significantly repressed in *E. faecalis* C1-1 (0.109-fold to 0.425-fold) (*p* < 0.05). Specifically, the DEG encoding an acetyl-CoA carboxylase (ACC) (*IUJ47_RS01530*) was significantly inhibited in *E. faecalis* C1-1 (0.109-fold) (*p* < 0.05). The ACC catalyzes the first committed step in fatty acid synthesis: the carboxylation of acetyl-CoA to form malonyl-CoA via a two-step reaction [56].

These results indicated that Fragment 1 from *C. communis* Linn. can inhibit the ribosome; phenylalanine, tyrosine, and tryptophan biosynthesis; carbon fixation pathways in prokaryotes; fatty acid biosynthesis; thiamine metabolism; and ABC transporters, which consequently leads to repressed cell membrane biosynthesis, energy storage, protein translation, and transport system in *E. faecalis* C1-1.

In order to verify the transcriptome data, we examined 30 representative DEGs by reverse-transcription real-time quantitative PCR (RT-qPCR) analysis, and the obtained data were generally consistent with the transcriptome results (Tables S1 and S2).

Overall, the comparative transcriptomic analyses revealed that Fragment 1 from *C. communs* Linn. can affect multiple metabolic pathways in Gram-positive *S. aureus* ATCC25923, *B. cereus* A1-1 and *E. faecalis* C1-1, as well as Gram-negative *S. enterica* ATCC15611. The ribosome, citrate cycle, oxidative phosphorylation, carbon fixation pathways in prokaryotes, purine metabolism, and nitrogen metabolism were significantly changed in certain Gram-positive and Gram-negative bacteria. On the other hand, the ABC transporters were inhibited in all the three Gram-positive bacteria, while some metabolic pathways were only altered in the Gram-negative bacterium, such as the glycolysis/gluconeogenesis, RNA degradation, pyruvate metabolism, and galactose metabolism. Additionally, the distinctly changed transcriptomic profiles were observed among the three Gram-positive bacteria. For instance, Fragment 1 from *C. communs* Linn. affected the propanoate metabolism, sulfur metabolism, cysteine and methionine metabolism, C5-branched dibasic acid metabolism, butanoate metabolism, and beta-lactam resistance in only *B. cereus* A1-1, whereas arginine biosynthesis, PPAR signaling pathway, and carotenoid biosynthesis were repressed in *S. aureus* ATCC25923. These results demonstrated that Fragment 1 from *C. communs* Linn. can repress energy metabolism in the four tested strains, hinder signal transmission, reduce the pumping capacity of exogenous harmful substances, thereby inhibiting bacterial growth and even leading to cell death.

## 3. Materials and Methods

### 3.1. Bacterial Strains and Culture Conditions

The bacterial strains and culture media used in this study are listed in Table S3. Each of the strains was inoculated into the corresponding media supplemented with 3.0% NaCl (pH 8.4–8.5, Vibrio strains) or 0.5/1.0% NaCl (pH 7.0–7.2, non-Vibrio strains), respectively, and incubated at 37 °C, as described in our previous reports [9,10]. Luria–Bertani (LB) and tryptic soy broth (TSB) media were purchased from Beijing Land Bridge Technology Co., Ltd., Beijing, China. Brain heart infusion (BHI) and Marine 2216 media were purchased from Qingdao Hope Bio-Technology Co., Ltd., Qingdao, China, and Becton, Dickinson and Company, New York, NY, USA, respectively.

### 3.2. Extraction of Bioactive Substances from C. communis Linn

The pharmacophagous plant *C. communis* Linn was purchased from the Caoxuan Food Store in Lishui City (27°25′37″ N, 118°41′28″ E) in Zhejiang Province, China in August 2020. The extraction of bioactive substances from *C. communis* Linn was carried out using the methanol and chloroform method described in our recent studies [9,10]. Briefly, an aliquot of 500 g fresh leaves and stems of *C. communis* Linn was freeze-dried at −80 °C for 48 h using the ALPHA 2-4 LD Plus Freeze-Dryer (Martin Christ, Osterode, Germany). The freeze-dried sample (10 g) was crushed, and then mixed with 99 mL chloroform:methanol (2:1, *v*/*v*) for 6 h, then subjected to ultrasonication using the Scientz IID ULtrasonic Cell Crusher (SCIENT Z, Ningbo, China) with the same parameters described previously [9]. The methanol phase and chloroform phase of the ultrasonic sample were separated and then subjected to rotary evaporation using a rotary evaporator (IKA, Staufen, Germany) to a viscous substance [9]. The chloroform and methanol (analytical grade) were purchased from Merck KGaA, Darmstadt, Germany.

### 3.3. Antimicrobial Susceptibility Assays

Antimicrobial susceptibility assays were performed according to a method described in our recent studies [9,10]. Blank disks (6 mm, Oxoid, Basingstoke, UK) and Mueller–Hinton (MH) were purchased from Oxoid, Basingstoke, UK. An aliquot of 10 μL crude extract (500 μg/mL) was added onto each disk and the bacteriostatic effect on the corresponding strains evaluated by measuring the diameter of the inhibition zone after incubation at 37 °C for 12 h. A gentamicin disk (10 μg, Oxoid, Basingstoke, UK) was used as a positive control, while the methanol phase with water and chloroform phase with anhydrous ethanol were used as negative controls.

Broth dilution testing (microdilution) was carried out to determine MICs of the extracts according to the standard method issued by the Clinical and Laboratory Standards Institute, USA (CLSI, M100-S28, 2018). The standard solution of gentamicin (100 µg/mL) was purchased from the National Standard Material Information Center, Beijing, China [9].

### 3.4. Prep-HPLC Analysis

The Prep-HPLC analysis was performed as described in our recent studies [9,10]. The extract sample (10 mg/mL) was dissolved in ultrapure water (analytical grade, Merck KGaA, Darmstadt, Germany), centrifugated at 8000× *g* for 20 min, and then passed through 0.22 μm membrane (Shanghai Sangon Biological Engineeing Technology and Service Co., Ltd., Shanghai, China). The filtration was subjected to Prep-HPLC using a Waters 2707 (Waters, Milford, MA, USA) linked with a UPLC Sunfire C18 column (5 μm, 10 × 250 mm) (Waters, Milford, MA, USA) with the parameters described recently by Liu et al. [9]. After the Prep-HPLC, the isolated fragments were individually collected, concentrated, and evaporated using the rotary evaporator (IKA, Staufen, Germany), and then redissolved in ultrapure water for further analyses.

### 3.5. Ultrahigh-Performance Liquid Chromatography–Mass Spectrometry (UHPLC–MS) Analysis

The UHPLC–MS analysis was conducted using the EXIONLC System (Sciex, Framingham, MA, USA) by Shanghai Hoogen Biotech, Shanghai, China, with the parameters as described previously [9,10]. The formic acid and acetonitrile (analytical grade) were purchased from Merck KGaA, Darmstadt, Germany. The SCIEX Analyst Work Station Software (version 1.6.3) was employed for multiple reaction monitoring (MRM) data acquisition and processing. In-house R program and database were applied for peak detection and annotation (Shanghai Hoogen Biotech, Shanghai, China) [9,10].

### 3.6. Scanning Electron Microscopy (SEM) Assay

Samples for SEM analysis were prepared according to the method described previously [9]. Briefly, 1 × MIC of Fragment 1 from *C. communis* Linn was added to bacterial culture (5 mL) grown to middle logarithmic growth phase (mid-LQP), and incubated at 37 °C for 2 h, 4 h and 6 h. From the cell suspension, 1.5 mL was collected, washed, fixed, and observed using an SU5000 scanning electron microscope (Hitachi, Tokyo, Japan, 5.0 kV, ×30,000) [10]. The untreated bacterial suspension was used as a negative control at 2 h, 4 h and 6 h.

### 3.7. Bacterial Cell Surface Hydrophobicity, Membrane Fluidity, and Cell Membrane Damage Assays

Bacterial cell surface hydrophobicity was determined according to the method of Araújo et al. [57]. Cell membrane fluidity was measured using the method of Flegler and Lipski [58]. Cetane and 1,6-diphenyl-1,3,5-hexatriene (DPH) were purchased from Shanghai Sangon Biological Engineeing Technology and Service Co., Ltd., Shanghai, China. Cell membrane damage was examined according to the method described by Yang et al. [59] using flow cytometry (BD FACSVerse; Becton, Dickinson and Company, New York, NY, USA).

### 3.8. Cell Membrane Permeability Analysis

Cell outer membrane permeability was determined according to the method of Baena-Santillán et al. [60]. Briefly, a 200 µL/well of bacterial cell suspension was mixed with 2 µL/well of 10 mm NPN solution (Shanghai Sangon Biological Engineeing Technology and Service Co., Ltd., Shanghai, China). The excitation and emission wavelengths were set at 350 nm and 420 nm, respectively, and recorded using BioTek Synergy 2 (BioTek, Burlington, VT, USA).

Cell inner membrane permeability was examined according to the method of Ibrahim et al. [61]. Briefly, a 200 µL/well of bacterial cell suspension was mixed with 2.5 µL/well of 10 mm ONPG solution (Shanghai Sangon Biological Engineeing Technology and Service Co., Ltd., Shanghai, China). The cell mixture was incubated at 37 °C and measured for each well at OD_415_ using BioTek Synergy 2 (BioTek, Burlington, VT, USA) every 30 min for 5 h.

### 3.9. Illumina RNA Sequencing

Bacteria strains grown to the mid-LGP were collected and treated with Fragment 1 from *C. communis* for 6 h. The untreated bacterial suspension was used as a negative control. Total RNA was prepared using an RNeasy Protect Bacteria Mini Kit (QIAGEN Biotech Co. Ltd., Hilden, Germany) and QIAGEN RNeasy Mini Kit (QIAGEN) according to the manufacturer’s protocols. DNA was removed from the samples using RNase-Free DNase Set (QIAGEN). Three independently prepared RNA samples were used for each Illumina RNA-sequencing analysis. The sequencing library construction and Illumina sequencing were conducted by Shanghai Majorbio Bio-pharm Technology Co. Ltd. (Shanghai, China) using an Illumina HiSeq 2500 platform (Illumina, Santiago, CA, USA). High-quality reads that passed the Illumina quality filters were used for sequence analyses [59].

### 3.10. RT-qPCR Assay

RT-qPCR was performed according to a method described previously [59]. The 16S rRNA gene was used as the internal reference gene, and 2^−ΔΔCt^ method was used to calculate the relative expression between the target and the internal reference genes. Oligonucleotide primers targeting 30 representative DEGs were designed (Table S1), and synthesized by Shanghai Sangon Biological Engineeing Technology and Service Co., Ltd., Shanghai, China.

### 3.11. Data Analysis

Expression of each gene was calculated using the RNA-Seq by Expectation-Maximization (RSEM, http://deweylab.github.io/RSEM/, accessed on 13 October 2022). The criteria for DEGs were the same as described in our precious report [59]. The DEGs were used for gene set enrichment analysis (GSEA) against the Kyoto Encyclopedia of Genes and Genomes (KEGG) database (http://www.genome.jp/kegg/, accessed on 13 October 2022). All the tests were conducted in triplicates. The data were analyzed using SPSS statistical analysis software version 17.0 (SPSS Inc., Chicago, IL, USA).

## 4. Conclusions

In this study, we analyzed antibacterial components in the methanol-phase extract from the traditional Chinese herb *C. communs* Linn for the first time. The antibacterial rate of the extract was 58.33% against 24 species of common pathogenic bacteria. The methanol-phase extract was further separated by Prep-HPLC, and four single fragments were obtained. Of these, Fragment 1 significantly increased cell surface hydrophobicity and membrane permeability, decreased membrane fluidity, and destroyed the cell membrane structure of Gram-positive bacteria including *B. cereus* A1-1, *E. faecalis* C1-1, and *S. aureus* ATCC25923, as well as Gram-negative bacterium *S. enterica* subsp. *enterica* (*ex* Kauffmann and Edwards) Le Minor and Popoff serovar Vellore ATCC15611. The damage to the bacterial cell membrane was conducive to the penetration of Fragment 1 through the cell surface, acting on the substances inside the cell. A total of 65 compounds with known functions in Fragment 1 were identified by UHPLC-MS, of which quercetin-3-o-glucuronide was predominant (19.35%). Comparative transcriptomic analysis revealed multiple altered metabolic pathways mediated by Fragment 1, such as inhibited ABC transporters, ribosome, citrate cycle and oxidative phosphorylation, and upregulated nitrogen metabolism and purine metabolism in the four representative bacterial strains (*p* < 0.05). These results demonstrated that Fragment 1 from *C. communs* Linn. can inhibit energy metabolism and protein translation, hinder signal transmission, and reduce the pumping capacity of exogenous harmful substances, thereby resulting in repressed bacterial growth and even death. Overall, the results of this study demonstrate that Fragment 1 from *C. communis* Linn is a promising candidate for pharmaceutical application against common pathogenic bacteria.

## Figures and Tables

**Table 1:** Antibacterial activity of the methanol-phase and chloroform-phase crude extracts from *C. communis* Linn.

Strain	Inhibition Zone (Diameter, mm)		MIC (μg/mL)	
	CPE	MPE	CPE	MPE
*Aeromonas hydrophila ATCC35654*	—	11.33 ± 1.24	—	128
*Bacillus cereus* A1-1	—	13.33 ± 0.94	—	64
*Enterobacter cloacae* ATCC13047	10.33 ± 0.47	—	512	—
*Enterobacter cloacae*	7.00 ± 0.00	7.63 ± 0.45	1024	1024
*Escherichia coli* ATCC8739	12.67 ± 0.47	—	128	—
*Escherichia coli* ATCC25922	12.00 ± 0.82	—	128	—
*Escherichia coli* K12	10.97 ± 0.78	—	256	—
*Enterococcus faecalis* C1-1	8.50 ± 0.41	18.03 ± 0.12	1024	32
*Enterobacter sakazakii* CMCC45401	—	—	—	—
*Listeria monocytogenes* ATCC19115	—	—	—	—
*Pseudomonas aeruginosa* ATCC9027	—	—	—	—
*Pseudomonas aeruginosa* ATCC27853	—	10.33 ± 0.47	—	256
*Salmonella enterica* subsp. *enterica* (*ex* Kauffmann and Edwards) Le Minor and Popoff serovar Choleraesuis ATCC13312	—	9.53 ± 0.41	—	256
*Salmonella paratyphi-A* CMCC50093	9.00 ± 0.00	—	1024	—
*Salmonella enterica* subsp. *enterica* (*ex* Kauffmann and Edwards) Le Minor and Popoff serovar Vellore ATCC15611	—	16.17 ± 0.39	—	32
*Salmonella* spp.	12.33 ± 1.25	12.20 ± 0.59	128	128
*Shigella dysenteriae* CMCC51252	9.00 ± 0.00	9.27 ± 0.54	1024	523
*Shigella flexneri* CMCC51572	13.33 ± 1.70	9.70 ± 0.29	64	512
*Shigella flexneri* ATCC12022	—	7.27 ± 0.21	—	1024
*Shigella flexneri* CMCC51574	—	—	—	—
*Shigella sonnei* ATCC25931	9.67 ± 0.94	—	512	—
*Shigella sonnet* CMCC51592	9.40 ± 0.29	—	512	—
*Staphylococcus aureus* ATCC25923	—	11.97 ± 0.82	—	128
*Staphylococcus aureus* ATCC8095	—	11.67 ± 0.47	—	128
*Staphylococcus aureus* ATCC29213	—	11.00 ± 0.82	—	256
*Staphylococcus aureus* ATCC6538	—	10.50 ± 0.41	—	256
*Staphylococcus aureus* ATCC6538P	—	8.90 ± 0.14	—	512
*Staphylococcus aureus*	7.00 ± 0.00	8.20 ± 0.28	1024	1024
*Vibrio alginolyticus* ATCC17749	11.67 ± 0.94	13.00 ± 0.00	256	128
*Vibrio alginolyticus* ATCC33787	—	—	—	—
*Vibrio cholerae* Q10-54	—	7.10 ± 0.08	—	1024
*Vibrio cholerae* b10-49	—	9.77 ± 0.88	—	256
*Vibrio cholerae* GIM1.449	—	10.53 ± 0.41	—	256
*Vibrio fluvialis* ATCC33809	—	—	—	—
*Vibrio harvey* ATCC BAA-1117	—	—	—	—
*Vibrio harveyi* ATCC33842	—	—	—	—
*Vibrio metschnikovii* ATCC700040	—	13.03 ± 0.32	—	64
*Vibrio mimicus* bio-56759	9.00 ± 0.00	—	512	—
*Vibrio parahaemolyticus* B3-13	—	9.00 ± 0.12	—	512
*Vibrio parahaemolyticus* B4-10	7.00 ± 0.00	—	1024	—
*Vibrio parahaemolyticus* B5-29	8.00 ± 0.00	17.67 ± 2.05	1024	32
*Vibrio parahaemolyticus* B9-35	—	8.67 ± 0.94	—	512
*Vibrio parahaemolyticus* ATCC17802	—	—	—	—
*Vibrio parahaemolyticus* ATCC33847	—	12.33 ± 1.25	—	128
*Vibrio vulnificus* ATCC27562	—	—	—	—

Note: CPE: chloroform-phase extract. MPE: methanol-phase extract. —: no bacteriostasis activity. Inhibition zone: diameter includes the disk diameter (6 mm). MIC: minimum inhibitory concentration. Values presented as means ± standard deviation (S.D.) of three parallel measurements.

**Table 2:** Antibacterial activity of Fragment 1 of the methanol-phase extract from *C. communis* Linn.

Bacterial Strain	Inhibition Zone (Diameter, mm)	MIC (μg/mL)
*B. cereus* A1-1	9.43 ± 0.17	512
*E. faecalis* C1-1	9.23 ± 0.25	512
*S. aureus* ATCC25923	10.37 ± 0.09	256
*S. aureus* ATCC8095	7.93 ± 0.12	512
*S. enterica* subsp. *enterica* (*ex* Kauffmann and Edwards) Le Minor and Popoff serovar Vellore ATCC15611	11.33 ± 0.25	128
*V. alginolyticus* ATCC17749	7.17 ± 0.09	1024
*V. metschnikovii* ATCC700040	7.30 ± 0.16	1024
*V. parahaemolyticus* B5-29	7.67 ± 0.17	1024

**Table 3:** The major compounds identified in Fragment 1 from *C. communis* Linn by the UHPLC–MS analysis.

Fragment No.	Identified Compound	Compound Nature	Rt (min)	Formula	Exact Mass	Fragment Area (%)
1	Quercetin-3-O-glucuronide	Flavonoids	5.92	C_21_H_18_O_13_	478.08	19.35
2	Glutamine	Amino acids	0.64	C_6_H_14_N_2_O_2_	146.11	8.69
3	Sucrose	Carbohydrates	0.89	C_12_H_22_O_11_	342.12	6.46
4	Methyl gallate	Phenols	4.44	C_8_H_8_O_5_	184.04	4.93
5	Indole	Alkaloids	3.82	C_8_H_7_N	117.06	4.52
6	cis-Aconitic acid	Organic acids	1.46	C_6_H_6_O_6_	174.02	2.46
7	Trigonelline	Alkaloids	0.82	C_7_H_7_NO_2_	137.05	2.32
8	Demethoxycurcumin	Phenols	4.91	C_20_H_18_O_5_	338.12	2.18
9	Campesterol	Steroids	12.18	C_28_H_48_O	400.37	2.16
10	2-Hydroxybutanoic acid	Organic acids	0.64	C_4_H_8_O_3_	104.05	2.04
11	Histidine	Amino acids	0.59	C_6_H_9_N_3_O_2_	155.07	1.91
12	L-Asparagine	Amino acids	0.64	C_4_H_8_N_2_O_3_	132.05	1.83
13	Maltol	Pyranones	0.90	C_6_H_6_O_3_	126.03	1.76
14	Nicotinamide	Alkaloids	1.01	C_6_H_6_N_2_O	122.05	1.76
15	Kaurenoic acid	Diterpenoids	5.90	C_20_H_3_0O_2_	302.22	1.53
16	Serine	Amino acid	0.62	C_3_H_7_NO_3_	105.04	1.48
17	22-Dehydroclerosterol	Steroids	12.59	C_29_H_46_O	410.35	1.43
18	Threonine	Amino acids	0.64	C_4_H_9_NO_3_	119.06	1.31
19	Homoserine	Amino acids	0.67	C_4_H_9_NO_3_	119.06	1.29
20	Miltirone	Diterpenoids	12.98	C_19_H_22_O_2_	282.16	1.23
21	Kirenol	Diterpenoids	13.16	C_20_H_34_O_4_	338.25	1.17
22	Denudatine	Alkaloids	1.59	C_22_H_33_NO_2_	343.25	1.17
23	4-Aminobutanoic acid	Organic acids and derivatives	0.66	C_4_H_9_NO_2_	103.06	1.17
24	Gingerol	Phenols	11.99	C_17_H_26_O_4_	294.18	1.16
25	Cinchonidine	Alkaloids	11.99	C_19_H_22_N_2O_	294.17	1.16
26	Proline	Amino acids	0.73	C_5_H_9_NO_2_	115.06	1.13
27	2-Picolinic acid	Organic acids	1.33	C_6_H_5_NO_2_	123.03	1.11
28	gamma-Diasarone	Lignans	8.55	C_24_H_32_O_6_	416.22	1.07
29	Stigmasterol	Steroids and steroid derivatives	13.12	C_29_H_48_O	412.37	0.93
30	Myrcene	Monoterpenoids	11.74	C_10_H_16_	136.13	0.93
31	Lumichrome	Alkaloids	6.70	C_12_H_10_N_4_O_2_	242.08	0.87
32	Demethoxyencecalin	Phenols	11.80	C_13_H_14_O_2_	202.10	0.87
33	Broussonin C	Phenols	13.23	C_20_H_24_O_3_	312.17	0.7
34	Maltotetraose	Carbohydrates	0.64	C_24_H_42_O_21_	666.22	0.6
35	Ethyl salicylate	Phenols	5.72	C_9_H_10_O_3_	166.06	0.58
36	Shionone	Triterpenoids	12.00	C_30_H_50_O	426.39	0.58
37	Betaine	Amino acid derivatives	1.06	C_5_H_11_NO_2_	117.08	0.58
38	Tacrolimus	Macrolide antibiotics	12.40	C_44_H_69_NO_12_	803.48	0.57
39	Hamamelitannin	Phenols	12.29	C_20_H_20_O_14_	484.09	0.56
40	1-Naphthylacetic acid	Phytohormones	10.55	C_12_H_10_O_2_	186.07	0.56
41	Tricin	Flavonoids	9.31	C_17_H_14_O_7_	330.07	0.55
42	L-Aspartic acid	Amino acids	0.63	C_4_H_7_NO_4_	133.04	0.55
43	Chamazulene	lipids	8.74	C_14_H_16_	184.13	0.55
44	Dihydromethysticin	Phenols	11.97	C_15_H_16_O_5_	276.10	0.55
45	Beta-Sitosterol	Steroids and steroid derivatives	12.93	C_29_H_50_O	414.39	0.54
46	Sterebin A	Diterpenoids	11.75	C_18_H_30_O_4_	310.21	0.53
47	Terpinine-4-ol	Monoterpenoids	5.45	C_10_H_18_O	154.14	0.53
48	Tangeretin	Flavonoids	11.37	C_20_H_20_O_7_	372.12	0.53
49	L-Homoglutamic acid	Amino acid and derivatives	0.73	C_6_H_11_NO_4_	161.07	0.53
50	Indole-3-carboxaldehyde	Indole alkaloids	6.73	C_9_H_7_NO	145.05	0.53
51	Moracin C	Phenols	13.99	C_19_H_18_O_4_	310.12	0.53
52	Ethyl phenylacetate	Phenols	2.43	C_10_H_12_O_2_	164.08	0.47
53	Carvacryl acetate	Monoterpenoids	11.23	C_12_H_16_O_2_	192.12	0.47
54	Cinchonine	Alkaloids	11.99	C_19_H_22_N_2O_	294.17	0.47
55	Kaempferitrin	Flavonoids	5.69	C_27_H_30_O_14_	578.16	0.43
56	Talatisamine	Alkaloids	4.66	C_24_H_39_NO_5_	421.28	0.43
57	Sciadopitysin	Flavonoids	12.82	C_33_H_24_O_10_	580.14	0.42
58	Physalin D	Steroids and steroid derivatives	12.10	C_28_H_32_O_11_	544.19	0.42
59	Morusin	Flavonoids	4.90	C_25_H_24_O_6_	420.16	0.42
60	Synephrine	Alkaloids	2.63	C_9_H_13_NO_2_	167.09	0.42
61	Citronellyl acetate	Monoterpenoids	10.48	C_12_H_22_O_2_	198.16	0.41
62	Exemestane	Steroids and steroid derivatives	11.61	C_20_H_24_O_2_	296.18	0.41
63	N-Methylcoclaurine	Alkaloids	12.07	C_18_H_21_NO_3_	299.15	0.41
64	Mevastatin	Statins/Esters	12.07	C_23_H_34_O_5_	390.24	0.39
65	Terpinolene	Monoterpenoids	10.57	C_10_H_16_	136.13	0.39

**Table 4:** The major altered metabolic pathways in *S. enterica* subsp. *enterica* (*ex* Kauffmann and Edwards) Le Minor and Popoff serovar Vellore ATCC15611 mediated by Fragment 1 from *C. communis* Linn.

Metabolic Pathway	Gene ID	Fold Change	Gene Description
Ribosome	SPC_3510	0.064	30S ribosomal protein S10
	SPC_3509	0.071	50S ribosomal protein L3
	SPC_3508	0.076	50S ribosomal protein L4
	SPC_2784	0.095	50S ribosomal protein L19
	SPC_3505	0.13	30S ribosomal protein S19
	SPC_2787	0.135	30S ribosomal protein S16
	SPC_3415	0.156	50S ribosomal protein L13
	SPC_3414	0.179	30S ribosomal protein S9
	SPC_3503	0.233	30S ribosomal protein S3
	SPC_3926	0.238	50S ribosomal protein L34
	SPC_3285	0.263	30S ribosomal protein S21
	SPC_3372	0.306	50S ribosomal protein L27
	SPC_3373	0.326	50S ribosomal protein L21
	SPC_3502	0.336	50S ribosomal protein L16
	SPC_4538	0.34	30S ribosomal protein S6
	SPC_3500	0.365	30S ribosomal protein S17
	SPC_3501	0.449	50S ribosomal protein L29
	SPC_3982	0.484	50S ribosomal protein L1
	SPC_0980	4.229	30S ribosomal protein S1
Citrate cycle	SPC_2263	0.084	Fumarate hydratase class I aerobic
	SPC_0731	0.097	Succinate dehydrogenase catalytic subunit
	SPC_0733	0.107	2-oxoglutarate dehydrogenase E1 component
	SPC_0729	0.111	Succinate dehydrogenase cytochrome b556 large membrane subunit
	SPC_2506	0.119	Isocitrate dehydrogenase
	SPC_0735	0.217	Succinyl-CoA synthetase subunit beta
	SPC_0734	0.227	Dihydrolipoamide acetyltransferase
	SPC_0736	0.25	Succinyl-CoA synthetase alpha subunit
	SPC_3569	0.296	Phosphoenolpyruvate carboxykinase
	SPC_0727	0.5	Citrate synthase
	SPC_0164	3.325	Dihydrolipoamide acetyltransferase
	SPC_0163	4.714	Pyruvate dehydrogenase E1 component
Glycolysis/Gluconeogenesis	SPC_3760	0.06	Aldehyde dehydrogenase B
	SPC_4566	0.241	Fructose-1-bisphosphatase
	SPC_4282	0.417	Glucose-6-phosphate isomerase
	SPC_1226	2.146	Glucose-specific PTS system enzyme IIA component
	SPC_4187	2.296	Triosephosphate isomerase
	SPC_1980	2.76	Alcohol dehydrogenase
	SPC_2451	3.023	Glyceraldehyde-3-phosphate dehydrogenase
	SPC_3111	3.382	6-phospho-beta-glucosidase
	SPC_2543	5.68	PTS system glucose-specific IIBC component
Oxidative phosphorylation	SPC_0730	0.057	Succinate dehydrogenase cytochrome b556 small membrane subunit
	SPC_1381	0.345	NADH dehydrogenase alpha subunit
	SPC_4565	0.409	Inorganic pyrophosphatase
	SPC_1383	0.475	NADH dehydrogenase I chain 2CD
	SPC_1387	0.497	NADH dehydrogenase subunit H
	SPC_0742	2.606	Cytochrome d ubiquinol oxidase subunit II
	SPC_0452	2.622	Protoheme IX farnesyltransferase
	SPC_0741	3.068	Cytochrome d ubiquinol oxidase subunit I
	SPC_0453	3.211	Cytochrome o ubiquinol oxidase C subunit
	SPC_0743	4.919	YbgT
	SPC_0454	5.338	Cytochrome o ubiquinol oxidase subunit III
	SPC_0456	6.012	Cytochrome o ubiquinol oxidase subunit II
	SPC_0455	8.594	Cytochrome o ubiquinol oxidase subunit I
Carbon fixation pathways	SPC_4339	0.047	Acetyl-coenzyme A synthetase
in prokaryotes	SPC_0732	0.127	Succinate dehydrogenase catalytic subunit
	SPC_1369	0.225	Phosphate acetyltransferase
	SPC_1370	0.335	Acetate/propionate kinase
	SPC_0170	0.417	Aconitate hydratase
RNA degradation	SPC_3353	0.421	Polynucleotide phosphorylase
	SPC_3062	0.421	Dinucleoside polyphosphate hydrolase
	SPC_4030	3.288	Transcription termination factor Rho
	SPC_3351	4.323	ATP-dependent RNA helicase
Purine metabolism	SPC_0328	0.151	Xanthine phosphoribosyltransferase
	SPC_1145	0.173	Bifunctional GMP synthase/glutamine amidotransferase protein
	SPC_4706	0.279	Purine nucleoside phosphorylase
	SPC_2977	0.281	Sulfate adenylyltransferase subunit 1
	SPC_2978	0.281	Sulfate adenylyltransferase subunit 2
	SPC_1144	0.295	Inositol-5-monophosphate dehydrogenase
	SPC_0502	0.454	Adenylate kinase
	SPC_1952	0.483	Ribose-phosphate pyrophosphokinase
	SPC_4695	2.018	Nucleotidase
	SPC_4513	2.067	Adenylosuccinate synthetase
	SPC_4705	2.177	Phosphopentomutase
	SPC_1128	2.354	Nucleoside diphosphate kinase
	SPC_4049	2.702	Adenylate cyclase
	SPC_4007	2.997	IMP cyclohydrolase
	SPC_3824	3.447	(P)ppGpp synthetase II/guanosine-5′-bis pyrophosphate 3′-pyrophosphohydrolase
	SPC_3822	3.463	Guanylate kinase
	SPC_1172	5.94	Phosphoribosylaminoimidazole-succinocarboxamide synthase
	SPC_4584	23.21	Anaerobic ribonucleoside triphosphate reductase
Methane metabolism	SPC_1369	0.225	Phosphate acetyltransferase
	SPC_2381	0.33	Phosphoenolpyruvate synthase
	SPC_4138	0.434	Formate dehydrogenase-O gamma subunit
	SPC_0768	2.857	Phosphoglyceromutase
Alanine, aspartate and	SPC_4476	0.089	Aspartate ammonia-lyase
glutamate metabolism	SPC_0071	0.234	Carbamoyl-phosphate synthase small subunit
	SPC_0072	0.351	Carbamoyl-phosphate synthase large subunit
	SPC_4110	0.489	Glutamine synthetase
	SPC_1377	3.031	Aspartate aminotransferase
	SPC_0689	4.495	Asparagine synthetase B
	SPC_3962	9.168	Asparagine synthetase AsnA
Nitrogen metabolism	SPC_3400	0.3	Glutamate synthase (NADPH) large chain precursor
	SPC_3401	0.421	Glutamate synthase (NADPH) small chain
	SPC_2160	3.204	Respiratory nitrate reductase 2 beta chain
	SPC_3544	6.181	Nitrite reductase large subunit
	SPC_2161	28.095	Respiratory nitrate reductase 2 alpha chain
	SPC_0939	29.218	Hydroxylamine reductase
Pyruvate metabolism	SPC_3776	0.04	Putative L-lactate dehydrogenase
	SPC_1189	0.475	Phosphate acetyltransferase
	SPC_0937	2.023	Pyruvate dehydrogenase
	SPC_0998	3.184	YcbL
Galactose metabolism	SPC_4361	0.009	Alpha-galactosidase
	SPC_0771	0.051	Galactose-1-phosphate uridylyltransferase
	SPC_0772	0.087	UDP-galactose 4-epimerase
	SPC_3916	0.199	2-oxo-3-deoxygalactonate kinase
	SPC_3914	0.397	Galactonate dehydratase
	SPC_2404	2.237	6-phosphofructokinase isozyme
	SPC_1977	2.93	Glucose-1-phosphate uridylyltransferase
	SPC_1252	32.911	Glucokinase

**Table 5:** The major altered metabolic pathways in *S. aureus* ATCC25923 mediated by Fragment 1 from *C. communis* Linn.

Metabolic Pathway	Gene ID	Fold Change	Gene Description
Oxidative	SAOUHSC_02340	0.249	ATP synthase F1 epsilon subunit
phosphorylation	SAOUHSC_00999	0.268	Quinol oxidase subunit IV putative
	SAOUHSC_01000	0.284	Cytochrome c oxidase subunit III putative
	SAOUHSC_01001	0.298	Quinol oxidase subunit I
	SAOUHSC_01002	0.350	Quinol oxidase AA3 subunit II putative
	SAOUHSC_01032	2.422	Cytochrome d ubiquinol oxidase subunit II putative
	SAOUHSC_01031	2.624	Cytochrome d ubiquinol oxidase subunit I putative
Valine, leucine and	SAOUHSC_02287	0.132	3-isopropylmalate dehydratase large subunit
isoleucine biosynthesis	SAOUHSC_02285	0.141	2-isopropylmalate synthase
	SAOUHSC_02286	0.142	3-isopropylmalate dehydrogenase
	SAOUHSC_02288	0.146	3-isopropylmalate dehydratase small subunit
	SAOUHSC_02289	0.156	Threonine dehydratase putative
	SAOUHSC_02284	0.172	Ketol-acid reductoisomerase
	SAOUHSC_02283	0.184	Conserved hypothetical protein
	SAOUHSC_02282	0.208	Acetolactate synthase large subunit biosynthetic type
	SAOUHSC_02281	0.331	Dihydroxy-acid dehydratase
ABC transporters	SAOUHSC_00634	0.012	ABC transporter substrate-binding protein putative
	SAOUHSC_00636	0.014	Iron (chelated) ABC transporter permease protein putative
	SAOUHSC_00637	0.015	Conserved hypothetical protein
	SAOUHSC_01945	0.095	Membrane protein putative
	SAOUHSC_00927	0.133	Oligopeptide ABC transporter substrate-binding protein putative
	SAOUHSC_00926	0.147	Oligopeptide ABC transporter ATP-binding protein putative
	SAOUHSC_00925	0.168	Conserved hypothetical protein
	SAOUHSC_02741	0.228	Amino acid ABC transporter permease protein putative
	SAOUHSC_00924	0.266	Conserved hypothetical protein
	SAOUHSC_02742	0.312	ABC transporter ATP-binding protein putative
	SAOUHSC_00668	0.316	ABC transporter permease putative
	SAOUHSC_00104	0.329	Amino acid ABC transporter ATP-binding protein putative
	SAOUHSC_02763	0.349	Peptide ABC transporter ATP-binding protein putative
	SAOUHSC_00923	0.355	Conserved hypothetical protein
	SAOUHSC_00240	0.356	Ribose ABC transporter protein putative
	SAOUHSC_00844	0.403	Conserved hypothetical protein
	SAOUHSC_00102	0.414	Phosphonates ABC transporter permease protein CC0363 putative
	SAOUHSC_00103	0.422	Phosphonates ABC transporter permease protein CC0363 putative
	SAOUHSC_02954	0.437	ABC transporter ATP-binding protein putative
	SAOUHSC_02743	0.444	Amino acid ABC transporter permease protein putative
	SAOUHSC_02719	0.472	Conserved hypothetical protein
	SAOUHSC_00843	0.474	Conserved hypothetical protein
	SAOUHSC_02953	0.480	Permease putative domain protein
	SAOUHSC_02718	0.483	Conserved hypothetical protein
	SAOUHSC_00424	2.156	ABC transporter permease protein putative
	SAOUHSC_02690	2.161	Conserved hypothetical protein
	SAOUHSC_02699	2.762	Conserved hypothetical protein
	SAOUHSC_02697	2.799	Amino acid ABC transporter ATP-binding protein putative
	SAOUHSC_02698	2.883	Amino acid ABC transporter permease protein putative
	SAOUHSC_00176	2.892	Bacterial extracellular solute-binding protein putative
	SAOUHSC_00423	3.536	Conserved hypothetical protein
	SAOUHSC_02430	3.658	ABC transporter periplasmic binding protein putative
	SAOUHSC_01656	4.262	Conserved hypothetical protein
	SAOUHSC_01657	4.964	ABC transporter putative
	SAOUHSC_00175	10.497	Multiple sugar-binding transport ATP-binding protein putative
	SAOUHSC_02640	22.861	Conserved hypothetical protein
	SAOUHSC_02641	37.417	Permease putative domain protein
Carotenoid biosynthesis	SAOUHSC_02879	0.222	Squalene desaturase putative
	SAOUHSC_02877	0.321	Squalene synthase putative
	SAOUHSC_02880	0.210	Conserved hypothetical protein
PPAR signaling pathway	SAOUHSC_01276	0.337	Glycerol kinase
	SAOUHSC_00198	0.492	Conserved hypothetical protein
	SAOUHSC_02860	3.217	HMG-CoA synthase putative
Ribosome	SAOUHSC_01191	0.258	Ribosomal protein L28
	SAOUHSC_02493	0.326	Ribosomal protein L30
	SAOUHSC_02494	0.353	Ribosomal protein S5
	SAOUHSC_02499	0.358	Ribosomal protein S14p/S29e putative
	SAOUHSC_02504	0.360	Ribosomal protein L29
	SAOUHSC_02492	0.369	Ribosomal protein L15
	SAOUHSC_02498	0.383	Ribosomal protein S8 putative
	SAOUHSC_02487	0.383	Conserved hypothetical protein
	SAOUHSC_02495	0.384	Ribosomal protein L18
	SAOUHSC_02496	0.389	Ribosomal protein L6 putative
	SAOUHSC_02488	0.391	Ribosomal protein L36
	SAOUHSC_02486	0.400	Ribosomal protein S11 putative
	SAOUHSC_02501	0.409	Ribosomal protein L24
	SAOUHSC_02502	0.413	Ribosomal protein L14
	SAOUHSC_02500	0.416	50S ribosomal protein L5 putative
	SAOUHSC_00017	0.419	Ribosomal protein L9
	SAOUHSC_02484	0.432	Ribosomal protein L17
	SAOUHSC_02503	0.442	30S ribosomal protein S17 putative
	SAOUHSC_02505	0.447	Ribosomal protein L16
	SAOUHSC_02506	0.469	Ribosomal protein S3
	SAOUHSC_02508	0.494	Ribosomal protein S19
Arginine biosynthesis	SAOUHSC_01128	0.246	Ornithine carbamoyltransferase
	SAOUHSC_01129	0.301	Carbamate kinase
	SAOUHSC_00148	0.335	Glutamate N-acetyltransferase/amino-acid acetyltransferase
	SAOUHSC_00147	0.388	Acetylglutamate kinase putative
	SAOUHSC_02134	0.397	Nitric oxide synthase oxygenase domain putative
	SAOUHSC_01287	2.119	Glutamine synthetase type I
	SAOUHSC_02409	2.658	Arginase
	SAOUHSC_02965	3.951	Carbamate kinase
	SAOUHSC_02968	4.068	Ornithine carbamoyltransferase
	SAOUHSC_02969	4.664	Arginine deiminase

**Table 6:** The major altered metabolic pathways in *B. cereus* A1-1 mediated by Fragment 1 from *C. communis* Linn.

Metabolic Pathway	Gene ID	Fold Change	Gene Description
Valine, leucine and	BCN_1374	0.158	Branched-chain amino acid aminotransferase
isoleucine biosynthesis	BCN_1381	0.230	3-isopropylmalate dehydratase small subunit
	BCN_1780	0.234	Dihydroxy-acid dehydratase
	BCN_1377	0.290	Ketol-acid reductoisomerase
	BCN_1378	0.314	2-isopropylmalate synthase
	BCN_1779	0.338	Ketol-acid reductoisomerase
	BCN_1781	0.356	Threonine dehydratase biosynthetic
	BCN_1379	0.402	3-isopropylmalate dehydrogenase
	BCN_4078	0.415	Leucine dehydrogenase
	BCN_2381	0.493	Threonine dehydratase catabolic
	BCN_0865	5.981	Acetolactate synthase
Beta-lactam resistance	BCN_0187	0.050	Oligopeptide ABC transporter oligopeptide-binding protein
	BCN_0190	0.057	Oligopeptide ABC transporter oligopeptide-binding protein putative
	BCN_3395	0.064	Oligopeptide ABC transporter oligopeptide-binding protein putative
	BCN_1762	0.069	Oligopeptide-binding protein
	BCN_2708	0.090	Oligopeptide ABC transporter oligopeptide-binding protein putative
	BCN_3396	0.115	Oligopeptide ABC transporter oligopeptide-binding protein
	BCN_1046	0.119	Penicillin-binding protein
	BCN_1433	0.132	Penicillin-binding protein
	BCN_1159	0.207	Oligopeptide ABC transporter permease protein
	BCN_1158	0.209	Oligopeptide ABC transporter oligopeptide-binding protein
	BCN_1956	0.261	Oligopeptide ABC transporter oligopeptide-binding protein putative
	BCN_1796	0.304	Oligopeptide ABC transporter oligopeptide-binding protein
	BCN_1160	0.306	Oligopeptide ABC transporter permease protein
	BCN_2267	0.319	Penicillin-binding protein
	BCN_1161	0.392	Oligopeptide ABC transporter ATP-binding protein
	BCN_1533	0.395	Penicillin-binding protein
	BCN_2412	0.402	Beta-lactamase
	BCN_1162	0.491	Oligopeptide ABC transporter ATP-binding protein
ABC transporters	BCN_1843	0.184	Branched-chain amino acid ABC transporter branched chain amino acid-binding protein putative
	BCN_3503	0.188	ABC transporter ATP-binding/permease protein
	BCN_0645	0.198	Ribose ABC transporter permease protein
	BCN_0174	0.204	ABC transporter substrate-binding protein putative
	BCN_0308	0.205	ABC transporter ATP-binding protein
	BCN_4185	0.207	Phosphate ABC transporter phosphate-binding protein
	BCN_4879	0.216	ABC transporter substrate-binding protein putative
	BCN_0173	0.228	ABC transporter ATP-binding protein
	BCN_0172	0.228	ABC transporter permease protein
	BCN_1262	0.259	Spermidine/putrescine ABC transporter permease protein
	BCN_1261	0.270	Spermidine/putrescine ABC transporter permease protein
	BCN_2821	0.271	Ribose ABC transporter permease protein putative
	BCN_0855	0.278	Amino acid ABC transporter permease protein
	BCN_0617	0.279	Amino acid ABC transporter amino acid-binding protein
	BCN_0852	0.289	ABC transporter ATP-binding/permease protein
	BCN_0853	0.323	Multidrug resistance ABC transporter ATP-binding and permease protein
	BCN_3526	0.334	Phosphonate ABC transporter permease protein
	BCN_4182	0.339	Phosphate ABC transporter ATP-binding protein
	BCN_1950	0.352	Adhesion lipoprotein
	BCN_1679	0.022	ABC transporter substrate-binding protein putative
	BCN_0309	0.025	ABC transporter permease protein
	BCN_4748	0.028	ABC transporter permease protein
	BCN_2777	0.029	Sulfonate ABC transporter sulfonate-binding protein putative
	BCN_2523	0.030	Cobalt transport protein
	BCN_4749	0.036	ABC transporter protein
	BCN_1678	0.043	ABC transporter ATP-binding protein
	BCN_3528	0.047	Phosphonate ABC transporter phosphonate-binding protein putative
	BCN_1845	0.073	Branched-chain amino acid ABC transporter ATP-binding protein
	BCN_4183	0.089	Phosphate ABC transporter permease protein
	BCN_1847	0.107	Branched-chain amino acid ABC transporter permease protein
	BCN_4882	0.110	ABC transporter ATP-binding protein
	BCN_4880	0.115	ABC transporter substrate-binding protein putative
	BCN_4184	0.115	Phosphate ABC transporter permease protein
	BCN_3525	0.123	Phosphonate ABC transporter permease protein
	BCN_4881	0.134	ABC transporter permease protein
	BCN_0694	0.138	Phosphate ABC transporter permease protein putative
	BCN_0854	0.139	Amino acid ABC transporter amino acid-binding protein
	BCN_1844	0.142	Branched-chain amino acid ABC transporter ATP-binding protein
	BCN_1846	0.158	Branched-chain amino acid ABC transporter permease protein
	BCN_0310	0.166	ABC transporter substrate-binding protein putative
	BCN_0856	0.168	Amino acid ABC transporter ATP-binding protein
	BCN_3443	0.172	bioY family protein
	BCN_1263	0.360	Spermidine/putrescine ABC transporter spermidine/putrescine-binding protein
	BCN_4814	0.381	BioY family protein
	BCN_0646	0.385	Ribose ABC transporter ribose-binding protein
	BCN_2820	0.391	Sugar ABC transporter sugar-binding protein putative
	BCN_3527	0.398	Phosphonate ABC transporter ATP-binding protein
	BCN_1680	0.398	ABC transporter permease protein putative
	BCN_0695	0.404	Phosphate ABC transporter permease protein putative
	BCN_0693	0.409	Phosphate ABC transporter phosphate-binding protein putative
	BCN_1260	0.438	Spermidine/putrescine ABC transporter ATP-binding protein
	BCN_3918	2.319	Maltosaccharide ABC transporter maltosaccharide-binding protein putative
	BCN_3917	2.348	Maltosaccharide ABC transporter permease protein
	BCN_2646	2.730	Glycine betaine/L-proline ABC transporter permease protein
	BCN_3921	2.821	Sugar ABC transporter ATP-binding protein
	BCN_0139	3.187	ABC transporter ATP-binding protein
	BCN_0141	3.264	Cobalt transport protein
	BCN_0140	3.409	ABC transporter ATP-binding protein
	BCN_5182	3.551	Lipoprotein putative
	BCN_0705	3.997	ABC transporter permease protein putative
	BCN_0706	4.033	ABC transporter substrate-binding protein putative
	BCN_0704	4.128	ABC transporter ATP-binding protein
	BCN_4065	4.749	Amino acid ABC transporter ATP-binding protein
	BCN_1856	5.441	Transport ATP-binding protein CydC
	BCN_2647	5.698	Glycine betaine/L-proline ABC transporter ATP-binding protein
	BCN_1857	6.622	Transport ATP-binding protein CydD
	BCN_0197	9.458	Molybdenum ABC transporter permease protein
	BCN_0199	15.950	Molybdate-binding protein
Propanoate metabolism	BCN_3213	0.003	Oxidoreductase aldo/keto reductase family
	BCN_4976	0.005	Oxidoreductase keto reductase family
	BCN_2421	0.041	Acetyl-CoA carboxylase biotin carboxylase putative
	BCN_2422	0.041	Conserved hypothetical protein
	BCN_4073	0.066	Dihydrolipoamide acetyltransferase
	BCN_2426	0.083	Acetoacetyl-CoA synthase putative
	BCN_4075	0.123	3-methyl-2-oxobutanoate dehydrogenase alpha subunit
	BCN_2273	0.277	Carboxyvinyl-carboxyphosphonate phosphorylmutase
	BCN_4554	0.347	Acetyl-CoA synthetase putative
	BCN_2276	0.354	Methylmalonic acid semialdehyde dehydrogenase
	BCN_4572	0.363	Acetyl-CoA synthetase
	BCN_2272	0.385	MmgE protein
	BCN_0770	0.401	Alcohol dehydrogenase zinc-containing
	BCN_2224	0.457	CoA-transferase beta subunit
Sulfur metabolism	BCN_2778	0.017	Sulfonate ABC transporter ATP-binding protein putative
	BCN_4282	0.036	Cystathionine beta-lyase
	BCN_2776	0.047	Sulfonate ABC transporter permease protein putative
	BCN_2775	0.049	Alkanesulfonate monooxygenase
	BCN_1400	0.053	Sulfate adenylyltransferase
	BCN_4168	0.058	Cystathionine beta-lyase
	BCN_1399	0.069	Phosphoadenosine phosphosulfate reductase
	BCN_1401	0.076	Adenylylsulfate kinase
	BCN_4664	0.187	Conserved hypothetical protein
	BCN_4261	0.199	Conserved hypothetical protein
	BCN_5333	0.227	Homoserine O-succinyltransferase
	BCN_1764	0.261	Cysteine synthase A
	BCN_0065	0.268	Cysteine synthase A
	BCN_1558	0.282	Rhodanese-like domain protein
	BCN_0166	49.152	Conserved hypothetical protein
Cysteine and methionine	BCN_1887	0.005	Homoserine dehydrogenase
metabolism	BCN_4283	0.035	O-acetylserine lyase
	BCN_3941	0.056	5-methylthioribose kinase putative
	BCN_4169	0.061	Cystathionine beta-lyase
	BCN_4284	0.061	MTA/SAH nucleosidase
	BCN_3907	0.069	5-methyltetrahydropteroyltriglutamate--homocysteine methyltransferase
	BCN_3940	0.069	Translation initiation factor putative aIF-2BI family
	BCN_3951	0.074	5-methylthio-3-oxo-1-penten-1-diol dioxygenase putative
	BCN_4285	0.075	Conserved hypothetical protein
	BCN_3950	0.086	Class II aldolase/adducin domain protein
	BCN_3949	0.090	Hydrolase haloacid dehalogenase-like family
	BCN_4167	0.098	Homocysteine S-methyltransferase domain protein/methylenetetrahydrofolate reductase family protein
	BCN_3948	0.117	Ribulose bisphosphate carboxylase putative
	BCN_4481	0.123	S-adenosylmethionine decarboxylase proenzyme
	BCN_3947	0.132	Aminotransferase classes I and II
	BCN_4166	0.166	5-methyltetrahydrofolate--homocysteine methyltransferase
	BCN_4053	0.198	L-serine dehydratase iron-sulfur-dependent alpha subunit
	BCN_4676	0.199	S-adenosylmethionine synthetase
	BCN_5334	0.210	O-acetylhomoserine/O-acetylserine sulfhydrylase
	BCN_5298	0.210	Spermidine synthase
	BCN_0360	0.230	5-methylthioribose kinase putative
	BCN_0361	0.238	Translation initiation factor putative aIF-2BI family
	BCN_5332	0.248	Homoserine dehydrogenase
	BCN_4054	0.337	L-serine dehydratase iron-sulfur-dependent beta subunit
	BCN_3089	0.384	Phosphoserine aminotransferase
	BCN_4706	0.414	Autoinducer-2 production protein LuxS
	BCN_2350	0.433	Aspartate-semialdehyde dehydrogenase
	BCN_4802	0.460	Aminotransferase classes I and II
	BCN_3629	2.260	Aspartate-semialdehyde dehydrogenase
C5-branched dibasic acid	BCN_1376	0.187	Acetolactate synthase small subunit
metabolism	BCN_1375	0.232	Acetolactate synthase large subunit biosynthetic type
	BCN_3665	0.266	Succinyl-CoA synthase beta subunit
	BCN_1380	0.271	3-isopropylmalate dehydratase large subunit
	BCN_3664	0.302	Succinyl-CoA synthase alpha subunit
	BCN_1778	0.337	Acetolactate synthase large subunit biosynthetic type
	BCN_0866	5.952	Alpha-acetolactate decarboxylase
Butanoate metabolism	BCN_3930	0.123	Acetyl-CoA acetyltransferase
	BCN_5271	0.127	Acyl-CoA dehydrogenase
	BCN_1290	0.164	3-oxoacyl-(acyl-carrier-protein) reductase putative
	BCN_1291	0.206	Poly(R)-hydroxyalkanoic acid synthase class III PhaC subunit
	BCN_5273	0.221	3-hydroxyacyl-CoA dehydrogenase
	BCN_4420	0.267	Succinate dehydrogenase flavoprotein subunit
	BCN_1790	0.274	Acetyl-CoA hydrolase/transferase family protein
	BCN_4419	0.298	Succinate dehydrogenase iron-sulfur protein
	BCN_4905	0.362	3-hydroxyacyl-CoA dehydrogenase/enoyl-CoA hydratase/isomerase family protein
	BCN_4077	0.428	Butyrate kinase
	BCN_0650	5.744	Alcohol dehydrogenase zinc-containing
	BCN_4281	19.159	Aldehyde-alcohol dehydrogenase
	BCN_0486	72.637	Formate acetyltransferase
Purine metabolism	BCN_2749	0.073	Inosine-uridine preferring nucleoside hydrolase family protein
	BCN_0015	0.113	Deoxynucleoside kinase family protein
	BCN_0009	0.131	Inosine-5′-monophosphate dehydrogenase
	BCN_4039	0.150	3′-cyclic-nucleotide 2′-phosphodiesterase
	BCN_2983	0.153	5′-nucleotidase putative
	BCN_1328	0.186	Ribonucleoside-diphosphate reductase alpha subunit group I intron-containing
	BCN_4016	0.203	5′-nucleotidase family protein
	BCN_0016	0.230	Deoxynucleoside kinase family protein
	BCN_1327	0.232	Ribonucleoside-diphosphate reductase alpha subunit group I intron-containing
	BCN_3354	0.292	Inosine-uridine preferring nucleoside hydrolase family protein
	BCN_1329	0.297	Ribonucleoside-diphosphate reductase beta subunit
	BCN_0284	0.394	Phosphoribosylaminoimidazole carboxylase catalytic subunit
	BCN_4734	0.432	Hypoxanthine phosphoribosyltransferase
	BCN_3700	0.450	Guanylate kinase putative
	BCN_0047	0.495	Ribose-phosphate pyrophosphokinase
	BCN_4604	2.023	Conserved hypothetical protein
	BCN_0061	2.194	Hypoxanthine phosphoribosyltransferase
	BCN_0286	3.142	Adenylosuccinate lyase
	BCN_0285	3.320	Phosphoribosylaminoimidazole carboxylase ATPase subunit
	BCN_0131	3.761	Adenylate kinase
	BCN_0289	4.896	Phosphoribosylformylglycinamidine synthase I
	BCN_4500	5.073	Pyruvate kinase
	BCN_4256	5.189	Conserved hypothetical protein
	BCN_0288	5.224	Phosphoribosylformylglycinamidine synthase PurS protein
	BCN_0290	7.207	Phosphoribosylformylglycinamidine synthase II
	BCN_0291	9.699	Amidophosphoribosyltransferase
	BCN_3413	12.000	Anaerobic ribonucleoside-triphosphate reductase putative
	BCN_0292	13.193	Phosphoribosylformylglycinamidine cyclo-ligase
	BCN_0293	13.562	Phosphoribosylglycinamide formyltransferase
	BCN_0294	13.974	Phosphoribosylaminoimidazolecarboxamide formyltransferase/IMP cyclohydrolase
	BCN_0287	14.299	Phosphoribosylaminoimidazole-succinocarboxamide synthase
	BCN_1550	15.096	Xanthine phosphoribosyltransferase
	BCN_0296	15.341	Phosphoribosylamine-glycine ligase
Citrate cycle	BCN_2633	0.036	Dihydrolipoamide acetyltransferase
	BCN_2271	0.118	Citrate synthase MmgD
	BCN_3424	0.263	Aconitate hydratase 1
	BCN_1240	0.297	2-oxoglutarate dehydrogenase E1 component
	BCN_1239	0.301	2-oxoglutarate dehydrogenase E2 component dihydrolipoamide succinyltransferase
	BCN_4421	0.313	Succinate dehydrogenase cytochrome b558 subunit
	BCN_1707	2.102	Fumarate hydratase class II
	BCN_3870	12.766	Pyruvate dehydrogenase complex E3 component dihydrolipoamide dehydrogenase
	BCN_3873	13.564	Pyruvate dehydrogenase complex E1 component alpha subunit
	BCN_3871	14.281	Pyruvate dehydrogenase complex E2 component dihydrolipoamide acetyltransferase
Nitrogen metabolism	BCN_1402	0.085	Nitrite reductase
	BCN_1469	0.379	Glutamate dehydrogenase
	BCN_2097	2.155	Nitrate transporter
	BCN_0507	2.456	Glutamate synthase large subunit putative
	BCN_4708	2.504	Carbonic anhydrase prokaryotic type putative
	BCN_2086	2.843	Respiratory nitrate reductase alpha subunit
	BCN_2087	3.780	Respiratory nitrate reductase beta subunit
	BCN_2089	6.543	Respiratory nitrate reductase gamma subunit
	BCN_2105	7.628	Nitrite reductase [NAD(P)H] large subunit
	BCN_2104	8.415	Nitrite reductase [NAD(P)H] small subunit

**Table 7:** Major altered metabolic pathways in *E. faecalis* C1-1 mediated by Fragment 1 from *C. communis* Linn.

Metabolic Pathway	Gene ID	Fold Change	Gene Description
Ribosome	IUJ47_RS11970	0.021	50S ribosomal protein L19
	IUJ47_RS03805	0.073	50S ribosomal protein L34
	IUJ47_RS02840	0.086	50S ribosomal protein L28
	IUJ47_RS02635	0.111	30S ribosomal protein S4
	IUJ47_RS13890	0.118	30S ribosomal protein S2
	IUJ47_RS08865	0.166	Type B 50S ribosomal protein L31
	IUJ47_RS01035	0.166	50S ribosomal protein L11
	IUJ47_RS01030	0.185	50S ribosomal protein L1
	IUJ47_RS04565	0.189	50S ribosomal protein L29
	IUJ47_RS04640	0.195	50S ribosomal protein L36
	IUJ47_RS04530	0.201	50S ribosomal protein L4
	IUJ47_RS07500	0.222	50S ribosomal protein L35
	IUJ47_RS01420	0.226	50S ribosomal protein L33
	IUJ47_RS04645	0.229	30S ribosomal protein S13
	IUJ47_RS04540	0.233	50S ribosomal protein L2
	IUJ47_RS04560	0.233	50S ribosomal protein L16
	IUJ47_RS04520	0.233	30S ribosomal protein S10
	IUJ47_RS07505	0.237	50S ribosomal protein L20
	IUJ47_RS04555	0.24	30S ribosomal protein S3
	IUJ47_RS04650	0.245	30S ribosomal protein S11
	IUJ47_RS04545	0.248	30S ribosomal protein S19
	IUJ47_RS04550	0.252	50S ribosomal protein L22
	IUJ47_RS14095	0.252	30S ribosomal protein S20
	IUJ47_RS04585	0.259	50S ribosomal protein L5
	IUJ47_RS04535	0.26	50S ribosomal protein L23
	IUJ47_RS04590	0.261	Type Z 30S ribosomal protein S14
	IUJ47_RS04525	0.264	50S ribosomal protein L3
	IUJ47_RS01020	0.28	50S ribosomal protein L7/L12
	IUJ47_RS04580	0.287	50S ribosomal protein L24
	IUJ47_RS04660	0.287	50S ribosomal protein L17
	IUJ47_RS04570	0.287	30S ribosomal protein S17
	IUJ47_RS04595	0.288	30S ribosomal protein S8
	IUJ47_RS04490	0.296	30S ribosomal protein S12
	IUJ47_RS04600	0.298	50S ribosomal protein L6
	IUJ47_RS04495	0.301	30S ribosomal protein S7
	IUJ47_RS04575	0.305	50S ribosomal protein L14
	IUJ47_RS04620	0.312	50S ribosomal protein L15
	IUJ47_RS04605	0.331	50S ribosomal protein L18
	IUJ47_RS01025	0.344	50S ribosomal protein L10
	IUJ47_RS04615	0.351	50S ribosomal protein L30
	IUJ47_RS04610	0.353	30S ribosomal protein S5
	IUJ47_RS07790	0.471	50S ribosomal protein L21
	IUJ47_RS07070	2.015	50S ribosomal protein L25/general stress protein Ctc
	IUJ47_RS03860	4.258	50S ribosomal protein L9
	IUJ47_RS06135	15.154	50S ribosomal protein L32
	IUJ47_RS03240	24.038	30S ribosomal protein S14
	IUJ47_RS03245	25.083	50S ribosomal protein L33
Phenylalanine, tyrosine	IUJ47_RS10435	0.021	Shikimate dehydrogenase
and tryptophan	IUJ47_RS10440	0.066	3-deoxy-7-phosphoheptulonate synthase
biosynthesis	IUJ47_RS11275	0.151	Type I 3-dehydroquinate dehydratase
	IUJ47_RS10445	0.154	3-dehydroquinate synthase
	IUJ47_RS10455	0.227	Prephenate dehydrogenase
	IUJ47_RS10460	0.262	3-phosphoshikimate 1-carboxyvinyltransferase
	IUJ47_RS10465	0.278	Shikimate kinase
	IUJ47_RS10450	0.3	Chorismate synthase
	IUJ47_RS10470	0.304	Prephenate dehydratase
Carbon fixation	IUJ47_RS00315	0.096	Flavocytochrome c
pathways in prokaryotes	IUJ47_RS01530	0.109	Acetyl-CoA carboxylase
	IUJ47_RS01515	0.158	Acetyl-CoA carboxylase carboxyltransferase subunit beta
	IUJ47_RS12350	0.183	Acetate kinase
	IUJ47_RS01520	0.262	Acetyl-CoA carboxylase biotin carboxylase subunit
	IUJ47_RS01510	0.294	Acetyl-CoA carboxylase carboxyl transferase subunit alpha
	IUJ47_RS07835	0.325	Bifunctional methylenetetrahydrofolate dehydrogenase/methenyltetrahydrofolate cyclohydrolase
	IUJ47_RS07670	0.397	Phosphate acetyltransferase
	IUJ47_RS08070	0.425	Pyruvate phosphate dikinase
	IUJ47_RS11250	0.425	Formate--tetrahydrofolate ligase
	IUJ47_RS00325	3.984	Pyruvate:ferredoxin (flavodoxin) oxidoreductase
Fatty acid biosynthesis	IUJ47_RS01560	0.075	Ketoacyl-ACP synthase III
	IUJ47_RS01525	0.151	3-hydroxyacyl-ACP dehydratase FabZ
	IUJ47_RS01550	0.161	Enoyl-acyl-carrier-protein reductase FabK
	IUJ47_RS05020	0.176	Enoyl-ACP reductase FabI
	IUJ47_RS01540	0.183	3-oxoacyl-acyl-carrier-protein reductase
	IUJ47_RS05025	0.218	Beta-ketoacyl-ACP synthase II
	IUJ47_RS01535	0.225	Beta-ketoacyl-ACP synthase II
	IUJ47_RS05030	0.336	3-hydroxyacyl-ACP dehydratase FabZ
	IUJ47_RS01545	0.344	ACP S-malonyltransferase
Thiamine metabolism	IUJ47_RS01260	0.161	Thiaminase II
	IUJ47_RS01310	0.209	Hydroxyethylthiazole kinase
	IUJ47_RS02170	0.231	Alkaline phosphatase
	IUJ47_RS01300	0.234	Bifunctional hydroxymethylpyrimidine kinase/phosphomethylpyrimidine kinase
	IUJ47_RS02080	0.353	tRNA 4-thiouridine (8) synthase ThiI
	IUJ47_RS04630	0.355	Adenylate kinase
	IUJ47_RS05190	0.355	Cysteine desulfurase
	IUJ47_RS01305	0.417	Thiamine phosphate synthase
	IUJ47_RS02855	0.421	Ribosome small subunit-dependent GTPase A
ABC transporters	IUJ47_RS12795	0.002	substrate-binding protein
	IUJ47_RS12785	0.003	ATP-binding protein
	IUJ47_RS12790	0.008	ABC transporter permease
	IUJ47_RS07400	0.012	ABC transporter permease subunit
	IUJ47_RS13490	0.036	Carbohydrate ABC transporter permease
	IUJ47_RS13485	0.05	ABC transporter substrate-binding protein
	IUJ47_RS07460	0.097	Oligopeptide ABC transporter substrate-binding protein Opp1A
	IUJ47_RS11560	0.101	Peptide ABC transporter substrate-binding protein
	IUJ47_RS10585	0.109	ABC transporter ATP-binding protein/permease
	IUJ47_RS07395	0.116	Amino acid ABC transporter ATP-binding protein
	IUJ47_RS10590	0.124	ABC transporter ATP-binding protein/permease
	IUJ47_RS06845	0.129	Amino acid ABC transporter substrate-binding protein/permease
	IUJ47_RS07470	0.136	Oligopeptide ABC transporter permease Opp1B
	IUJ47_RS02495	0.157	Peptide ABC transporter substrate-binding protein
	IUJ47_RS07475	0.198	Oligopeptide ABC transporter permease Opp1C
	IUJ47_RS01265	0.211	Cobalt ABC transporter permease
	IUJ47_RS09160	0.212	ABC transporter permease subunit
	IUJ47_RS06840	0.218	Amino acid ABC transporter ATP-binding protein
	IUJ47_RS01680	0.22	ABC transporter ATP-binding protein
	IUJ47_RS03225	0.226	ABC transporter permease subunit
	IUJ47_RS11415	0.232	Phosphate ABC transporter substrate-binding protein PstS
	IUJ47_RS10195	0.232	Peptide ABC transporter substrate-binding protein
	IUJ47_RS08600	0.236	Amino acid ABC transporter permease
	IUJ47_RS11410	0.253	Phosphate ABC transporter permease subunit PstC
	IUJ47_RS11400	0.256	Phosphate ABC transporter ATP-binding protein PstB
	IUJ47_RS04720	0.265	ABC transporter permease subunit
	IUJ47_RS12860	0.279	Energy-coupling factor transporter transmembrane protein EcfT
	IUJ47_RS09165	0.282	Carbohydrate ABC transporter permease
	IUJ47_RS11395	0.285	Phosphate ABC transporter ATP-binding protein PstB
	IUJ47_RS04685	0.288	Energy-coupling factor transporter transmembrane protein EcfT
	IUJ47_RS01745	0.295	Multidrug efflux ABC transporter subunit EfrA
	IUJ47_RS10055	0.299	ABC-F type ribosomal protection protein
	IUJ47_RS08330	0.299	Peptide ABC transporter substrate-binding protein
	IUJ47_RS11150	0.324	Phosphate ABC transporter substrate-binding protein PstS family protein
	IUJ47_RS04095	0.33	Peptide ABC transporter substrate-binding protein
	IUJ47_RS11420	0.335	Permease-like cell division protein FtsX
	IUJ47_RS03230	0.346	Methionine ABC transporter ATP-binding protein
	IUJ47_RS03220	0.368	MetQ/NlpA family ABC transporter substrate-binding protein
	IUJ47_RS11425	0.368	Cell division ATP-binding protein FtsE
	IUJ47_RS09170	0.384	ABC transporter substrate-binding protein
	IUJ47_RS11405	0.39	Phosphate ABC transporter permease PstA
	IUJ47_RS02680	0.392	Peptide ABC transporter substrate-binding protein
	IUJ47_RS07480	0.405	Oligopeptide ABC transporter ATP-binding protein Opp1D
	IUJ47_RS11280	0.416	ABC transporter ATP-binding protein/permease
	IUJ47_RS08615	0.418	Amino acid ABC transporter ATP-binding protein
	IUJ47_RS04680	0.425	Energy-coupling factor ABC transporter ATP-binding protein
	IUJ47_RS12995	0.429	ABC transporter ATP-binding protein
	IUJ47_RS01740	0.447	Multidrug efflux ABC transporter subunit EfrB
	IUJ47_RS04675	0.451	Energy-coupling factor ABC transporter ATP-binding protein
	IUJ47_RS08610	0.464	Transporter substrate-binding domain-containing protein
	IUJ47_RS02640	0.471	Biotin transporter BioY
	IUJ47_RS04715	0.472	Amino acid ABC transporter ATP-binding protein
	IUJ47_RS00725	2.036	ABC transporter substrate-binding protein
	IUJ47_RS07265	2.115	Osmoprotectant ABC transporter substrate-binding protein
	IUJ47_RS07260	2.394	ABC transporter permease
	IUJ47_RS09700	2.483	Sugar ABC transporter permease
	IUJ47_RS09705	2.566	Extracellular solute-binding protein
	IUJ47_RS04400	2.715	BMP family protein
	IUJ47_RS12720	3.049	Thiol reductant ABC exporter subunit CydD
	IUJ47_RS04405	3.21	BMP family protein
	IUJ47_RS09695	3.237	Sugar ABC transporter permease
	IUJ47_RS04420	3.248	ABC transporter permease
	IUJ47_RS12715	3.422	Thiol reductant ABC exporter subunit CydC
	IUJ47_RS02110	8.113	D-ribose pyranase
	IUJ47_RS03260	40.433	ZinT/AdcA family metal-binding protein

## Data Availability

The complete lists of DEGs in the four strains are available in the NCBI SRA database (https://submit.ncbi.nlm.nih.gov/subs/bioproject/) under the accession number PRJNA890025.

## Supplementary Materials

The following supporting information can be downloaded at https://www.mdpi.com/article/10.3390/plants12040890/s1. Table S1: The oligonucleotide primers designed and used in the RT-qPCR assay; Table S2: The relative expression of representative DEGs in the RT-qPCR assay; Table S3: The bacterial strains and media used in this study; Figure S1: The Prep-HPLC diagram of purifying the methanol-phase extract from *C. communis* Linn.
