# Supplementary material for: Antibacterial Components and Modes of the Methanol-Phase Extract from Commelina communis Linn
Source: Plants (Basel). 2023 Feb 16;12(4):890. doi: 10.3390/plants12040890 (PMC9966474; doi:10.3390/plants12040890)
Supplement: Supplementary file 1 [file plants-12-00890-s001.zip › plants-2155032-supplementary.pdf]

plants

MDPI

Supplementary Materials

Table S1. The oligonucleotide primers designed and used in the RT-qPCR assay.

Bacterial Strain	Primer	Sequence (5′->3′)
B. cereus A1-1	*BCN_3213*-F	GTATCCTTGGTCTGCGTTC
	BCN_3213-R	GACCCTGATAACTTTGATTTCT
	BCN_1887-F	AGGGTATGAAGTGAAGGTGAA
	BCN_1887-R	CCCTACATTAACAACATTATTCAT
	BCN_2778-F	GCAAATGACTTCGGGTAAA
	BCN_2778-R	GGATTGATTCAAACAAGTTTATAG
	BCN_0296-F	ATACAGGTGGAATGGGTGC
	BCN_0296-R	GCGTCCAATTACTAAAACATTC
	BCN_4281-F	TATCTGTAATAACCGCAAATG
	BCN_4281-R	TGAAAGCATATAAAGGTGAGTA
	BCN_0166-F	AGCAGGTTTGGATTTACACTA
	BCN_0166-R	AAATTAAGCAAAACTGCGTAG
	BCN_0486-F	AGGTGTTATTACGGGTCTTC
	BCN_0486-R	AACATTTTCTAATACTTGAGTCAT
E. faecalis C1-1	*IUJ47_RS12795*-F	ACCTGAACAAATGACCACG
	IUJ47_RS12795-R	AGCAGCTAACGTTAACCCT
	IUJ47_RS12785-F	TGGTGCTGGAAAGTCAACA
	IUJ47_RS12785-R	GCTAAGTTAAAGCTTTTTCTCAT
	IUJ47_RS12790-F	TGTCTTTGATGGGTGATGC
	IUJ47_RS12790-R	CATCAATAAATGCAGCAATCA
	IUJ47_RS03240-F	GCTGGCGATTATGAAGGTC
	IUJ47_RS03240-R	CAATTTTTGATTTTTTAGCCAT
	IUJ47_RS03245-F	ACTGGCGAGCGCCTTTAC
	IUJ47_RS03245-R	ACGTAATGGTTTGGCGCA
	IUJ47_RS03260-F	GTAGGCGATGAAGGAGACG
	IUJ47_RS03260-R	CAGGGGAAGAGTAAATTTTTTC
S. aureus ATCC25923	*SAOUHSC_00634*-F	TGCCATTTGACTTATCACTGC
	SAOUHSC_00634-R	TGTACACGGAAGCATGAAATAA
	SAOUHSC_00636-F	AATAAGAATGATAGGGCAACAC
	SAOUHSC_00636-R	AAACGAGGAAGTTTAACATGA
	SAOUHSC_00637-F	AAACTTCACTTTCGGACATCA
	SAOUHSC_00637-R	CAAAGGAGCAATATAACATGTTAG
	SAOUHSC_01945-F	ATTGTTCTACCGCTCCATTT
	SAOUHSC_01945-R	GCGGTAGAACAATTAAAGATTAA
	SAOUHSC_00175-F	ATTACGAATGGTTGCTGGACT
	SAOUHSC_00175-R	TGCTCTAACTTTAGTTCTGCCAT
	SAOUHSC_02640-F	GCTCTCATAATCGCAACACG
	SAOUHSC_02640-R	GAAGATGGCAAAATAACTGATTAG

	SAOUHSC_02641-F	AACGGTGCTTGCTCTGCTT
	SAOUHSC_02641-R	GCAATTGGAGGTGCAGAATAA
S. enterica ATCC15611	*SPC_4339*-F	TTTTGGCAGTGAGTCGGTC
	SPC_4339-R	ATCGCGATGCCGTCATAA
	SPC_0730-F	CTTCATCCTGGTCCGTGCTA
	SPC_0730-R	ATGCGGAGGCGTTGCTTA
	SPC_3760-F	GCTCACCAGCAGGCACTT
	SPC_3760-R	ATAAGCCTCTGGGGCTGTT
	SPC_3510-F	GACGCATCAGAGCATCAAC
	SPC_3510-R	GCAGATCAGCCTGGGTTA
	SPC_3509-F	GTTACCGTGAGTAGCGTCTTG
	SPC_3509-R	ACCAGCTGTGAAGGCGTAA
	SPC_2543-F	TACATCGGCTACGCTCACG
	SPC_2543-R	GATGAGTACATCCGTAACAGCTA
	SPC_0455-F	TGCGGAATGGAAGACGAT
	SPC_0455-R	GGCTGAAAAATGGCAACTGA
	SPC_4584-F	CGCTTCACTTCTTCCTGCT
	SPC_4584-R	CAATGGGCAGATCGGTTAA
	SPC_0939-F	GCCTTCAAGATGGCTAACG
	SPC_0939-R	CAATTGCTGAGCGCGTAA
	SPC_1252-F	GTAGGAGATGTAGGCGGCA
	SPC_1252-R	CTCCTACTAAAGCATACTTTGTCA

Table S2. The relative expression of representative DEGs by the RT-qPCR assay.

Bacterial Strain	Gene	Predicted protein	Fold Change	
			**RNA-Seq.**	**RT-qPCR**
B. cereus A1-1	*BCN_3213*	Oxidoreductase aldo / keto reductase family	0.003	0.012
	BCN_1887	Homoserine dehydrogenase	0.005	0.011
	BCN_2778	Sulfonate ABC transporter ATP-binding protein putative	0.017	0.059
	BCN_0296	Phosphoribosylamine-glycine ligase	15.341	31.381
	BCN_4281	Aldehyde-alcohol dehydrogenase	19.159	21.362
	BCN_0166	Conserved hypothetical protein	49.152	82.437
	BCN_0486	Formate acetyltransferase	72.637	41.375
E. faecalis C1-1	*IUJ47_RS12795*	Substrate-binding protein	0.002	0.021
	IUJ47_RS12785	ATP-binding protein	0.003	0.006
	IUJ47_RS12790	ABC transporter permease	0.008	0.017
	IUJ47_RS03240	30S ribosomal protein S14	24.038	16.377
	IUJ47_RS03245	50S ribosomal protein L33	25.083	25.317
	IUJ47_RS03260	ZinT/AdcA family metal-binding protein	40.433	26.148
S. aureus ATCC25923	*SAOUHSC_00634*	ABC transporter substrate-binding protein putative	0.012	0.083
	SAOUHSC_00636	Iron (chelated) ABC transporter permease protein putative	0.014	0.146
	SAOUHSC_00637	Conserved hypothetical protein	0.015	0.157
	SAOUHSC_01945	Membrane protein putative	0.095	0.114
	SAOUHSC_00175	Multiple sugar-binding transport ATP-binding protein putative	10.497	20.977
	SAOUHSC_02640	Conserved hypothetical protein	22.861	41.365
	SAOUHSC_02641	Permease putative domain protein	37.417	13.291
S. enterica ATCC15611	*SPC_4339*	Acetyl-coenzyme A synthetase	0.047	0.092
	SPC_0730	Succinate dehydrogenase cytochrome b556 small membrane subunit	0.057	0.093
	SPC_3760	Aldehyde dehydrogenase B	0.06	0.132
	SPC_3510	30S ribosomal protein S10	0.064	0.084
	SPC_3509	50S ribosomal protein L3	0.071	0.101
	SPC_2543	PTS system glucose-specific IIBC component	5.68	6.833
	SPC_0455	Cytochrome o ubiquinol oxidase subunit I	8.594	7.434
	SPC_4584	Anaerobic ribonucleoside triphosphate reductase	23.21	11.86
	SPC_0939	Hydroxylamine reductase	29.218	31.027

SPC_1252	Glucokinase	32.911	31.288

Table S3. The bacterial strains and media used in this study.

Bacterial Strain	Culture Medium	Source
Vibrio alginolyticus ATCC17749	TSB	ATCC, United States
Vibrio alginolyticus ATCC33787	TSB	ATCC, United States
Vibrio fluvialis ATCC33809	Marine 2216	ATCC, United States
Vibrio harvey ATCC BAA-1117	Marine 2216	ATCC, United States
Vibrio harveyi ATCC33842	Marine 2216	ATCC, United States
Vibrio metschnikovii ATCC700040	Marine 2216	ATCC, United States
Vibrio mimicus bio-56759	TSB	Biobw, China
Vibrio parahaemolyticus ATCC17802	TSB	ATCC, United States
Vibrio parahaemolyticus ATCC33847	TSB	ATCC, United States
Vibrio vulnificus ATCC27562	TSB	Biobw, China
Aeromonas hydrophila ATCC35654	TSB	ATCC, United States
Bacillus cereus A1-1	TSB	LS-SHOU, China
Enterobacter cloacae ATCC13047	TSB	Biobw, China
Enterobacter cloacae	LB	LS-SHOU, China
Escherichia coli ATCC8739	TSB	Biobw, China
Escherichia coli K12	TSB	IIM, China
Escherichia coli ATCC25922	LB	ATCC, United States
Enterobacter sakazakii CMCC45401	TSB	Biobw, China
Enterococcus faecalis C1-1	TSB	LS-SHOU, China
Listeria monocytogenes ATCC19115	BHI	Biobw, China
Pseudomonas aeruginosa ATCC9027	TSB	Biobw, China
Pseudomonas aeruginosa ATCC27853	TSB	Biobw, China
Staphylococcus aureus ATCC 25923	TSB	ATCC, United States
Staphylococcus aureus ATCC 8095	TSB	ATCC, United States
Staphylococcus aureus ATCC29213	TSB	ATCC, United States
Staphylococcus aureus ATCC6538	TSB	ATCC, United States
Staphylococcus aureus ATCC6538P	TSB	ATCC, United States
Staphylococcus aureus	TSB	LS-SHOU, China
Shigella dysenteriae CMCC51252	TSB	GCCC, China
Salmonella enterica subsp. *enterica* (*ex* Kauffmann and Edwards) Le Minor and Popoff serovar Choleraesuis ATCC13312	TSB	ATCC, United States
Shigella flexneri CMCC51572	TSB	GCCC, China
Shigella flexneri ATCC12022	TSB	ATCC, United States
Shigella flexneri CMCC51574	TSB	GCCC, China
Salmonella paratyphi-A CMCC50093	TSB	GCCC, China
Shigella sonnei ATCC25931	TSB	ATCC, United States
Shigella sonnet CMCC51592	TSB	GCCC, China
Salmonellaenterica subsp. *enterica* (*ex* Kauffmann and Edwards) Le Minor and Popoff serovar Vellore ATCC15611	TSB	ATCC, United States
Salmonella	LB	LS-SHOU, China

Vibrio parahaemolyticus B3-13	TSB	LS-SHOU, China
Vibrio parahaemolyticus B4-10	TSB	LS-SHOU, China
Vibrio parahaemolyticus B5-29	TSB	LS-SHOU, China
Vibrio parahaemolyticus B9-35	TSB	LS-SHOU, China
Vibrio cholerae GIM1.449	TSB	GCCC, China
Vibrio cholerae Q10-54	TSB	LS-SHOU, China
Vibrio cholerae b10-49	TSB	LS-SHOU, China

ATCC: American Type Culture Collection, United States; GCCC, Guangdong Culture Collection Center, Guangzhou, China; IIM, Institute of Industrial Microbiology, Shanghai, China; LS-SHOU, Laboratory stock, Shanghai Ocean University, Shanghai, China.
